# Vegetarian dietary patterns and cardiovascular risk factors and disease prevention: An umbrella review of systematic reviews

**DOI:** 10.1016/j.ajpc.2024.100868

**Published:** 2024-09-28

**Authors:** Matthew J. Landry, Katelyn E. Senkus, A Reed Mangels, Nanci S. Guest, Roman Pawlak, Sudha Raj, Deepa Handu, Mary Rozga

**Affiliations:** a Department of Population Health and Disease Prevention, University of California, Program in Public Health, 856 Health Sciences Rd., Irvine, CA 92697, United States; b Department of Human Nutrition, The University of Alabama, Tuscaloosa, AL, United States; c USDA/ARS Children's Nutrition Research Center, Baylor College of Medicine, 1100 Bates Avenue, Houston, TX 77030, United States; d Retired, 190 Walnut Avenue, Santa Cruz, CA 95060, United States; e Department of Nutritional Science, Temerty Faculty of Medicine, University of Toronto, Toronto, ON, Canada; f Department of Nutrition Science, East Carolina University, Greenville, NC 27858, United States; g Department of Nutrition and Food Studies, David B Falk College of Sport and Human Dynamics, Syracuse University, Syracuse, NY 13214, United States; h Evidence Analysis Center, Academy of Nutrition and Dietetics, 120 S. Riverside Plaza, Suite 2190, Chicago, IL 60606-6995, United States

**Keywords:** Vegetarians, Vegans, Dietary patterns, Cardiovascular diseases, Systematic review, Umbrella review

## Abstract

**Background:**

Diet significantly influences the risk of developing cardiovascular disease (CVD), the leading cause of death in the United States. As vegetarian dietary patterns are increasingly being included within clinical practice guidelines, there is a need to review the most recent evidence regarding if and how these dietary patterns mitigate CVD risk.

**Objective:**

This umbrella review of systematic reviews compared the relationships between vegetarian, vegan and non-vegetarian dietary patterns and CVD health outcomes and risk factors among presumably healthy adults (≥18 years) in the general population.

**Methods:**

MEDLINE, CINAHL, Cochrane Databases of Systematic Reviews, Food Science Source and SportsDiscus databases were searched for systematic reviews (SRs) published from 2018 until March 2024. Eligible SRs and meta-analyses examined relationships between vegetarian or vegan diets and CVD risk factors and disease outcomes compared to non-vegetarian diets. SRs were screened in duplicate, and SR quality was assessed with AMSTAR2. The overall certainty of evidence (COE) was evaluated using the Grading of Recommendation, Assessment, Development, and Evaluation (GRADE) method.

**Results:**

There were 758 articles identified in the databases’ search and 21 SRs met inclusion criteria. SRs targeting the general population had primarily observational evidence. Vegetarian, including vegan, dietary patterns were associated with reduced risk for CVD incidence [Relative Risk: 0.85 (0.79, 0.92)] and CVD mortality [Hazard Ratio: 0.92 (0.85, 0.99)] compared to non-vegetarian diets. Vegan dietary patterns were associated with reductions in CVD risk factors including blood pressure [systolic mean difference (95 % CI): -2.56 mmHg (-4.66, -0.445)], low-density lipoprotein cholesterol [-0.49 mmol/l (-0.62, -0.36)], and body mass index [-1.72 kg/m^2^ (-2.30, -1.16)] compared to non-vegetarian dietary patterns, as well as c-reactive protein concentrations in a novel meta-analysis [-0.55 mg/l (-1.07, -0.03)].

**Conclusion:**

Practitioners can consider recommending vegetarian dietary patterns to reduce cardiometabolic risk factors and risk of CVD incidence and mortality.

## 1 Introduction

Cardiovascular disease (CVD) continues to pose a significant global health challenge, creating a considerable burden due to both morbidity and mortality. Prominent heart-health organizations, such as the American Heart Association and the American College of Cardiology, underscore the critical role of dietary modifications in the prevention and management of CVD [[1], [2], [3]]. Recommendations emphasize the significance of adopting dietary patterns that focus on reducing consumption of red and processed meats while concomitantly increasing the intake of plant-derived foods [1,2]. A comprehensive analysis of 78 clinical practice guidelines across the globe revealed that 49 % advocate for plant-focused dietary patterns [4].

A healthy vegetarian-style dietary pattern includes some degree of restriction of animal products (Supplementary Fig. 1 [5]) while being rich in vegetables and fruits, legumes, whole grains, nuts, and seeds [[6], [7], [8]]. Vegetarian-style dietary patterns are typically low in saturated fats and cholesterol while providing health-promoting foods rich in fiber, antioxidants, and phytochemicals. These dietary attributes are key factors in maintaining cardiovascular health, including improved lipid profiles, blood pressure (BP) regulation, enhancing endothelial function, inflammation control, as well as contributing to a diverse microbiome [[9], [10], [11], [12]]. However, a poorly planned vegetarian-style dietary pattern can limit specific nutrients such as vitamin B12, iron, and calcium or include an excess of refined carbohydrates, added sugar, or saturated fat which can contribute to increased CVD risk [7,13,14].

In recent years, there has been an emergence of numerous systematic reviews (SRs) on the impact of vegetarian dietary patterns for CVD risk and prevention. This underscores the growing recognition and scientific interest in the potential benefits of vegetarian dietary patterns for maintaining cardiovascular health. However, these SRs have variable quality and typically focus on limited outcomes. To support development and refinement of evidence-based dietary recommendations and clinical practice guidelines for primary prevention of CVD, a systematic and comprehensive overview of current evidence was warranted. Therefore, our objective was to conduct an umbrella review to (1) systematically identify, compare, and summarize relevant systematic reviews and meta-analyses on the relationships between vegetarian, vegan and non-vegetarian dietary patterns and prevention of cardiovascular risk factors and health outcomes among presumably healthy adults in the general population and (2) evaluate the quality/certainty of the best available evidence.

## 2 Methods

An overview of SRs, or an umbrella review, was conducted due to high availability of existing SRs examining the relationships between dietary patterns and outcomes of interest [15]. This umbrella review followed methods for Overviews of Reviews from the Cochrane Collaboration [16] and was reported using the Preferred Reporting Items for Systematic Reviews and Meta-Analyses (PRISMA) checklist [17]. Methods were specified *a priori* and registered at The International Prospective Register of Systematic Reviews (PROSPERO) (CRD42023396453) [18].

### 2.1 Eligibility criteria

Eligibility criteria are described in Table 1. Briefly, participants were required to be presumably healthy adults (≥18 years) in the general population. The exposure of interest was vegetarian including vegan dietary patterns, and the comparison of interest was non-vegetarian dietary patterns. The greater aim of this umbrella review was to inform health care professionals of the comprehensive benefits and potential risks of vegetarian and vegan dietary patterns, and, thus, several outcomes were assessed. This umbrella review is part of a greater project examining the impact of vegetarian dietary patterns [18]. This manuscript reports the outcomes for cardiovascular risk and disease, and results for other outcomes are available on the Academy of Nutrition and Dietetics Evidence Analysis Library website [19]. Primary outcomes of interest included CVD incidence, events and mortality. Coronary heart disease (CHD) and ischemic heart disease were both described as CHD. Secondary outcomes included hypertension and overweight/obesity incidence, BP, low-density lipoprotein (LDL) cholesterol and triglyceride (TG) concentrations, c-reactive protein (CRP) concentrations, and body mass index (BMI). Publication date was limited to SRs published since 2018 because older SRs do not include recent research. Included SRs were limited to peer-reviewed articles published in the English language due to resource constraints. No supplemental primary studies were included.

**Table 1:** Eligibility criteria for the research question examining the relationships between vegetarian, vegan and non-vegetarian diets in presumably healthy adults in the general population.

Category	Inclusion Criteria	Exclusion Criteria
**Population**	Human adults ≥18 years	<18 years of age, animal studies
**Health Status**	Studies targeting or with sub-group analysis of presumably healthy adults in the general population	Studies which *target* participants with limited generalizability to the general population, such as those with diagnosed • HTN, dyslipidemia, overweight/obesity, impaired glucose tolerance • Type 2 DM • CVD • Eating disorders • COPD • HIV/AIDS • Post-bariatric surgery • Severe/persistent mental illness • Pregnancy
**Intervention/ Exposure**	Vegetarian (including lacto- and lacto-ovo- vegetarians) and/or vegan diets and all sub-groups. Plant-based diets will only be included if the definition meets the definition of a vegetarian or vegan diet.	Diets not meeting vegetarian/vegan definition. Flexitarian, pescatarian, semi-vegetarian diets. Studies that do not define “plant-based” and in which it is not possible to determine if plant-based is being used to mean vegetarian/vegan. Samples that combine data for semi-vegetarians and/or pescatarians with vegetarians.
**Exposure Measurement**	For observational studies, participants may consume meat/poultry/fish ≤once/month and still be classified as “vegetarian”.	Not applicable
**Comparison**	Non-vegetarian diets. Vegan diets may serve as comparison for vegetarian diets.	• No comparison group. • The comparison group is an intervention that differs from the vegetarian/vegan diet in more ways than inclusion of meat, animal, products, etc.
**Outcome**	**Health Outcomes:** • Disease Incidence ○ CVD, HTN, CV Events, Overweight/Obesity • CVD Mortality **Intermediate Outcomes:** • BP • LDL-Cholesterol, TGs • CRP • BMI	Outcomes not specified in inclusion criteria
**Study Design**	Systematic Reviews and Meta-Analysis SRs can include RCTs, non-randomized controlled trials, longitudinal cohort studies and cross-sectional studies. SRs must address the research question and search at least two databases. SRs that are rated as having higher quality with the AMSTAR2 tool, those that conduct meta-analysis, those that assess risk of bias of individual articles and those with graded certainty of evidence will be prioritized for evidence synthesis.	Primary studies, case reports, narrative reviews, commentaries
**Study Duration**	No limits.	No limits.
**Sample Size**	No limits.	No limits.
**Publication Dates**	2018- March 6, 2024	Published prior to 2018 or after search date of March 6, 2024.
**Publication Status**	peer-reviewed publications	Gray literature, conference abstracts

**Abbreviations:** AMSTAR2= *A* MeaSurement Tool to Assess systematic Reviews version 2, BMI= body mass index; BP= blood pressure; COPD= chronic obstructive pulmonary disorder; CRP= C-reactive protein; CV= cardiovascular; CVD= cardiovascular disease; DM= diabetes mellitus; HIV/AIDS= Human immunodeficiency virus infection and acquired immune deficiency syndrome; HTN= hypertension; LDL= low-density lipoprotein; RCT= randomized controlled trial; SR= systematic review.

### 2.2 Information sources

The databases search was designed and conducted by an information specialist who searched MEDLINE, CINAHL, Cochrane Databases of Systematic Reviews, Food Science Source and SportsDiscus databases. The search included terms such as “vegetarian”, “vegan”, and “plant-based”, with limits on language (English language), publication date (2018-March 6, 2024) and study design (systematic reviews and meta-analyses). Full search strategies are described in Supplementary Table 1.

### 2.3 Selection process

Following de-duplication, titles and abstracts of identified articles were independently screened by two reviewers using Rayyan software [20]. After initial title/abstract screening, potentially included full-text SRs were assessed by two independent reviewers. Discrepancies in screening were settled through consensus by expert panel members. The study selection process was documented in a PRISMA flow diagram [17]. Overlap of primary studies included in SRs was addressed during data extraction and evidence summary.

### 2.4 Data collection

A standardized data extraction sheet was created to extract study characteristics for each SR. Data were extracted by trained evidence analysts and checked by a second reviewer. Data included bibliographic information, participant health status and ages, exposure/intervention details [e.g., vegetarian, vegan or non-vegetarian diet, diet duration], eligible primary study designs, date of published studies in SRs, whether meta-analysis was conducted, if risk of bias (RoB) was assessed and with which tool, if certainty of evidence (COE) was assessed and with which tool, and outcomes of interest reported.

### 2.5 Quality assessment

The quality of SRs was assessed by two independent reviewers with the A MeaSurement Tool to Assess systematic Reviews version 2 (AMSTAR2) tool [21,22]. Discrepancies in AMSTAR2 ratings were settled with consensus or a third reviewer. RoB of primary studies as assessed in included SRs were used for certainty of evidence (COE) assessment [16].

### 2.6 Synthesis methods

Characteristics of included SRs were described in a study characteristics table. When more than one SR examined the relationship between vegetarian dietary patterns and a specific outcome of interest, primary studies from each SR were plotted to determine overlap. Several of the SRs relied on previously conducted meta-analyses. Umbrella review authors analyzed study overlap according to primary studies, even if data from secondary studies was used in an included SR.

SRs with higher quality as assessed by the AMSTAR2 tool, those that conducted meta-analysis and/or assessed RoB and COE, and those that were more recent and comprehensive were prioritized when interpreting results. If only one SR conducted meta-analysis, umbrella review authors discussed if results from the other included SR(s) agreed. If more than one SR conducted a meta-analysis, umbrella review authors determined appropriateness of combining the data from both meta-analyses to increase coverage and statistical power. If data could not be combined, the effect estimate from the higher quality SR was prioritized.

When a novel meta-analysis could be conducted, data was extracted from the SRs, not the primary studies themselves [16]. When meta-analysis was conducted for this umbrella review, we estimated the effect size and 95 % CI using a DerSimonian-Laird random-effects model as true associations between vegetarian dietary patterns and outcomes may vary according to sub-population [23]. For continuous outcomes, results were reported as mean differences (MD) and 95 % confidence interval (CI). Results of meta-analysis were visualized using forest plots. Publication bias was assessed using funnel plots and Egger's statistics. Heterogeneity was measured using the I^2^ measure. Sub-group analyses were conducted for each research question regarding the type of vegetarian dietary pattern and the RoB of primary studies. Statistical analyses were conducted using OpenMeta [24] and RStudio [25]. A p-value of <0.05 was considered statistically significant.

### 2.7 Certainty of evidence assessment

Umbrella review authors used the Grading of Recommendation, Assessment, Development, and Evaluation (GRADE) method to assess COE [26]. If authors of included SRs graded COE, ratings between the umbrella review and SR were compared and any deviations from the SR grading were justified. A summary of findings table was drafted using gradePRO [27], which reports the number of studies and participants, effect size, factors contributing to COE and overall conclusion for each outcome and was adapted for this manuscript.

## 3 Results

**Fig. 1:**
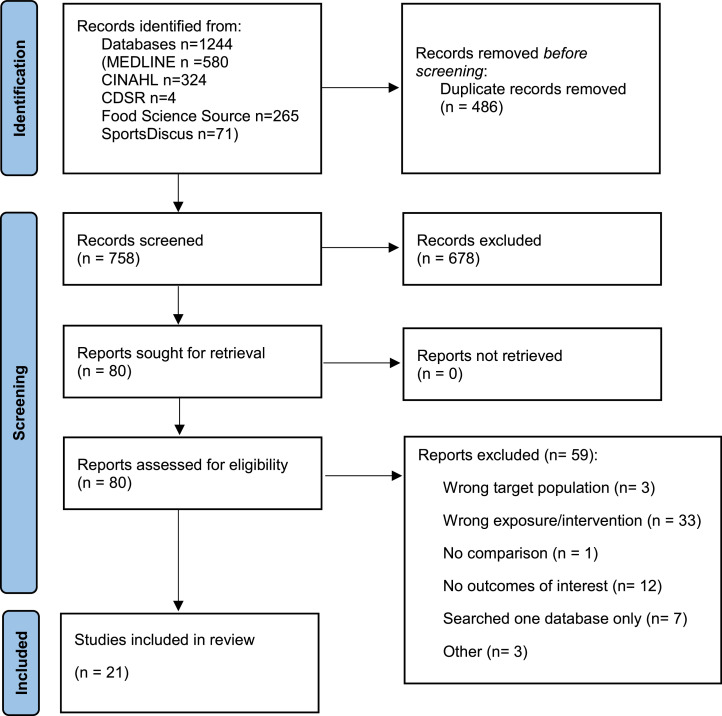
PRISMA flow diagram for new systematic reviews which included searches of databases and registers only.

Database searches identified 758 SRs; we screened 80 full-text SRs, and 21 SRs were included (Fig. 1) [[28], [29], [30], [31], [32], [33], [34], [35], [36], [37], [38], [39], [40], [41], [42], [43], [44], [45], [46], [47], [48]]. A list of articles excluded during full text review and reasons is available in Supplementary Table 2. Characteristics of included studies are described in Table 2. Seven SRs examined vegetarian and vegan dietary patterns combined [30,34,36,41,44,47,48], six SRs examined vegan dietary patterns [28,29,38,40,43,45], and eight SRs provided separate results for both vegetarian and vegan dietary patterns [[31], [32], [33],^35^,^37^,^39^,^42^,^46^]. Quality of SRs as assessed by the AMSTAR2 tool are shown in Supplementary Table 3. Though several SRs aimed to describe the impact of dietary patterns in the general population using RCTs, generally the RCTs available examined adults with cardiometabolic disease only. Thus, despite intending to include SRs with RCTs, umbrella review authors judged that these SRs often did not answer the research question, as studies targeting adults with cardiometabolic disease may have different goals, evaluation of efficacy and comparison diets compared to those evaluating impact for the general population. Thus, conclusions were primarily based on results from observational evidence. For most outcomes, included SRs did not report data required to combine data from different SRs in a novel meta-analysis, except for CRP concentrations, for which a novel meta-analysis was conducted.

**Table 2:** Study characteristics of included systematic reviews examining the relationship between vegetarian and vegan diets and cardiovascular risk and disease outcomes.

Author, Year	Population (Health Status, Age)	Diet (Vegetarian, Vegan) Diet Duration	Study Designs Included	Years of Primary Studies	Diet Adherence Assessment Reported	Outcomes of Interest Reported	Meta-Analysis	RoB Assessment	Certainty of Evidence Grading	Overall Confidence in Results from AMSTAR2
Bakaloudi et al. 2021 [28]	No limits on health status or age	Vegan Duration: NA-11.7 years	Cross-sectional, cohort	NR	Yes	BMI	No	Newcastle-Ottawa and modified Newcastle-Ottawa Scale	No	Low
Benatar et al. 2018 [29]	No significant comorbidities ≥18 years	Vegan Duration: ≥1 year	Cross-sectional, cohort	1960–2018	Yes	• BMI • BP • LDL-C • TGs	Yes	Newcastle-Ottawa Scale	No	Critically Low
Craddock et al. 2019 [30]	No limits on health status or age	Vegetarian Duration: ≥4 weeks for interventions, ≥1 year for observational	Cross-sectional, RCTs	NA-2017	No	CRP	Yes	Modified Newcastle-Ottawa Scale	GRADE	Critically Low
Dybvik et al. 2022 [31]	Presumably healthy general population No limits on age	Vegetarian, Vegan Duration: 5.14–28.3 years	Prospective Cohort Studies	NA-2021	No	• CVD Incidence • CVD Mortality • IHD Incidence • IHD Mortality • Stroke Incidence • Stroke Mortality	Yes	Modified ROBINS-I	World Cancer Research Fund grading criteria	Low
Elliot et al. 2022 [32]	No limits on health status ≥18 years	Vegetarian, Vegan Duration: 3 months- 3.75 years	RCT, prospective cohort, cross-sectional	NA-2022	No	• LDL-C • TGs • CRP/ hsCRP	No	No	No	Critically Low
Gibbs et al. 2021 [33]	No limits on health status ≥18 years	Vegetarian, Vegan Duration: 1.4–204 weeks (median 12 weeks)	Controlled trials	1961–2019	No	BP	Yes	RoB2	GRADE	Low
Glenn et al. 2019 [34]	With or without diabetes Median age range: 33–58 years	Vegetarian Duration: ≥1 year	Prospective cohort	NA-2018	Yes	• CHD Incidence • CHD Mortality • CVD Mortality • Stroke Mortality	Yes	Newcastle-Ottawa	GRADE	Critically Low
Ivanova et al. 2021 [35]	No limits on health status ≥18 years	Vegetarian, Vegan Duration: 30 days-2 years	Controlled trials, prospective cohort studies	NA-2021	No	• BMI • LDL-C	No	Newcastle-Ottawa Scale	No	Critically Low
Jabri et al. 2021 [36]	No limits on health status or age	Vegetarian Duration: Mean of 10.68 years	Prospective Cohort Studies	NA-2019	No	• CHD Mortality • CBVD Mortality	Yes	ROBINS-I	GRADE	Critically Low
Jafari et al. 2021 [37]	No limit on health status ≥18 years	Vegetarian, Vegan Duration: 4–25 years	Prospective Cohort Studies	1988–2020	Yes	• CVD Mortality • CBVD Mortality • CHD Mortality	Yes	Newcastle-Ottawa	GRADE	Low
Kaiser et al. 2021 [38]	No limits on health status or age	Vegan Duration 8 weeks-18.1 years	RCTs, Prospective Cohort, Cross-sectional	NR-2020	Yes	• Stroke Incidence • Myocardial Infarction Incidence • CHD Incidence • CHD Incidence • CVD Mortality • CBVD Mortality	No	Cochrane Collaboration Tool and Newcastle-Ottawa	Agency for Healthcare Research and Quality	Low
Koch et al. 2023 [47]	No limit on health status ≥18 years	Vegetarian, Vegan	RCTs	1980–2022	No	LDL-C TGs	Yes	RoB 2	No	Critically Low
Lee et al. 2020 [39]	No limits on health status or age	Vegetarian, Vegan Duration: ≥ 2 weeks	RCTs	NA-2020	No	BP	Yes	RoB	GRADE	Critically Low
Lopez et al. 2019 [40]	No limit on health status ≥18 years	Vegan Duration: ≥3 weeks	RCTs	NA-2018	No	BP	Yes	NR	GRADE	Low
Lu et al. 2021 [41]	No history of stroke No limits on age	Vegetarian Duration: 6–26 years	Prospective Cohort Studies	NA-2021	Yes	Stroke Incidence	Yes	Newcastle-Ottawa	Nutrigrade	High
Menzel et al. 2020 [42]	No limit on health status ≥18 years	Vegetarian, Vegan Duration: 1–25 years	Cross-sectional	NA-2020	No	CRP	Yes	Newcastle-Ottawa Quality Assessment Scale adapted for cross-sectional studies	No	Low
Pollakova et al. 2021 [43]	Healthy ≥18 years	Vegan Duration: 6–74 weeks	RCTs	NR-2020	No	BMI	No	Cochrane Risk of Bias tool	No	Critically Low
Quek et al. 2021 [44]	No limit on health status 38–67 years	Vegetarian Duration: NR	Prospective Cohort Studies	NA-2021	Yes	• CVD Incidence • CVD Mortality • Stroke Incidence	Yes	Newcastle-Ottawa	No	Critically Low
Rees et al. 2021 [45]	General Population or with CVD Risk	Vegan Duration: 8–39 weeks	RCTs	1900–2020	No	• CVD Events • CVD Mortality • LDL-C • TGs • BP • BMI	Yes	RoB2	GRADE (for some outcomes)	High
Remde et al. 2022 [46]	No limit on health status Adult	Vegetarian, Vegan Duration: ≥4 weeks	RCTs and Quasi-experimental studies	NR-2020	No	• BMI • BP • LDL-C • TGs	No	Cochrane RoB	No	Low
Wang et al. 2023 [48]	No limit on health status ≥18 years	Vegetarian Duration: 2–36 years	Prospective cohort studies, case-cohort studies, or nested case-control studies	NR- 2023	Yes	• CVD Incidence • T2DM Incidence	Yes	Quality Assessment Tool for Observational Cohort and Cross-Sectional Studies	No	Critically Low

**Abbreviations:** AMSTAR2= *A* MeaSurement Tool to Assess systematic Reviews version 2, BMI= body mass index, BP= blood pressure, CBVD= cerebrovascular disease, CRP= C-reactive protein, CHD= coronary heart disease, CVD= cardiovascular disease, GRADE= Grading of Recommendations, Assessment, Development, and Evaluations, IHD= ischemic heart disease, hsCRP= high-sensitivity c-reactive protein, LDL-C= low-density lipoprotein cholesterol, NA= not applicable, NR= not reported, RCT= randomized controlled trial, ROB= risk of bias, RoB2= Version 2 of the Cochrane risk-of-bias tool for randomized trials, ROBINS-*I*= Risk Of Bias In Non-Randomized Studies - of Interventions; TGs= triglycerides.

### 3.1 Cardiovascular disease and coronary heart disease incidence

Five SRs examined the relationship between consuming vegetarian dietary patterns, compared to non-vegetarian dietary patterns, and CVD and CHD incidence in presumably healthy adults in the general population [31,34,38,44,48]. Among these, Dybvik et al. [31] and Kaiser et al. [38] had the highest quality as assessed by the AMSTAR2 tool (Supplementary Table 3). In total, nine primary studies were included in the SRs and eight of these studies were reported in Dybvik et al. [31].

In the SR by Dybvik et al. [31], follow-up ranged from 5.14 to 28.3 years. In meta-analysis, vegetarian including vegan dietary patterns reduced risk of both CVD [relative risk (RR) (95 % CI): 0.85 (0.79, 0.92) I^2^=68 %] and CHD [0.79 (0.71, 0.88) I^2^=67 %] compared to non-vegetarian dietary patterns (Table 3). In a meta-analysis of six studies in Dybvik et al. [31], there was no significant relationship between consuming vegan dietary patterns, compared to non-vegetarian dietary patterns, and CVD [0.92 (0.79, 1.06) I^2^= 0 %] or CHD [0.82 (0.68, 1.00) I^2^= 0 %] incidence (Table 3). It is important to note that Dybvik et al. [31]. reported incidence by using data on CVD and CHD incidence when available and using CVD and CHD mortality as a measure of incidence (i.e., CVD/CHD must have been incident if participants died of this cause) when incidence was not available. Dybvik et al. [31] rated vegetarian including vegan dietary patterns as having a “probably protective effect”. Authors described a “limited-suggestive” impact of vegan dietary patterns on CHD, and “limited-no conclusion” for the impact of vegan dietary patterns on CVD [31].

**Table 3:** Quantitative results of included systematic reviews examining the impact of vegetarian diets compared to non-vegetarian diets on cardiovascular disease (CVD) incidence and results for presumably healthy adults in the general population.

Author, Year	Diet	Outcome	N Studies	N Participants	Effect Size Type	Effect Size	95 % CI	I^2^	CoE^1^	SR Quality^3^
**CVD Incidence**										
Dybvik et al. 2022 [31]	Vegetarian	CVD Incidence or Mortality^1^	8	621,282	RR	0.85	0.79, 0.92	68 %	Probably protective effect	Low
Quek et al. 2021[44]	Vegetarian	CVD Incidence	1	16,254	HR	0.81	0.72, 0.91	NA	NR	Critically Low
Dybvik et al. 2022 [31]	Vegan	CVD Incidence or Mortality^1^	6	197,668	RR	0.92	0.79, 1.06	0 %	Limited-no conclusion	Low
Dybvik et al. 2022 [31]	Vegetarian	CHD^2^ Incidence or Mortality^1^	8	621,282	RR	0.79	0.71, 0.88	67 %	Probably protective effect	Low
Glenn et al. 2019 [34]	Vegetarian	CHD Incidence	1	44,561	RR	0.72	0.61, 0.85	NA	Very Low	Critically Low
Wang et al. 2023	Vegetarian	CVD Incidence^4^	5	Unclear	RR	0.85	0.70, 1.03	99 %	NR	Critically Low
Dybvik et al. 2022 [31]	Vegan	CHD Incidence or Mortality^1^	6	197,668	RR	0.82	0.68, 1.00	0 %	Limited-suggestive	Low
Kaiser et al. 2021 [38]	Vegan	CHD Incidence	1	26,260	HR	0.82	0.64, 1.05	NA	Low	Low
**Cardiovascular Events**										
Dybvik et al. 2022 [31]	Vegetarian	Total Stroke	12	770,867	RR	0.90	0.77, 1.05	61 %	Limited-no conclusion	Low
Quek et al. 2021 [44]	Vegetarian	Total Stroke	Unclear	20,397	HR	0.72	0.36, 1.41	81.9 %	NR	Critically Low
Lu et al. 2021 [41]	Vegetarian	Total Stroke	7	679,773	HR	0.86	0.67, 1.11	68 %	Low	High
Dybvik et al. 2022 [31]	Vegan	Total Stroke	5	109,938	RR	1.17	0.69, 1.99	28 %	Limited-no conclusion	Low
Kaiser et al. 2021 [38]	Vegan	Total Stroke	1	26,260	HR	1.35	0.95, 1.92	NA	Very Low	Low
Kaiser et al. 2021 [38]	Vegan	Myocardial Infarction	1	26,260	HR	0.77	0.46, 1.27	NA	NR	Low
**CVD Mortality**										
Glenn et al. 2019 [34]	Vegetarian	CVD Mortality	5	144,247	RR	0.92	0.84, 1.02	34 %	Very Low	Critically Low
Jafari et al. 2021 [37]	Vegetarian	CVD Mortality	5	144,247	HR	0.92	0.85, 0.99	0 %	NR Vegetarian Diets	Low
Quek et al. 2021 [44]	Vegetarian	CVD Mortality	3	58,274	HR	0.89	0.78, 1.01	0 %	NR	Critically Low
Jafari et al. 2021 [37]	Lacto-ovo-vegetarian	CVD Mortality	1	73,308	HR	0.90	0.76, 1.06	NA	NR Vegetarian Diets	Low
Jafari et al. 2021 [37]	Vegan	CVD Mortality	1	73,308	HR	0.91	0.71, 1.16	NA	NR Vegetarian Diets	Low
Kaiser et al. 2021 [38]	Vegan	CVD Mortality	1	40, 907	HR	0.91	0.71, 1.16	NA	NR	Low
Glenn et al. 2019 [34]	Vegetarian	CHD Mortality	7	197,737	RR	0.78	0.69, 0.88	46 %	Very Low	Critically Low
Jabri et al. 2021 [36]	Vegetarian	CHD Mortality	7	127,517	RR	0.70	0.55, 0.89	82 %	Very Low	Critically Low
Jafari et al. 2021 [37]	Vegetarian	CHD Mortality	7	197,377	HR	0.76	0.68, 0.85	35 %	NR Vegetarian Diets	Low
Jafari et al. 2021 [37]	Lacto-ovo-vegetarian	CHD Mortality	1	Unclear	HR	0.82	0.63, 1.07	NA	NR Vegetarian Diets	Low
Jafari et al. 2021 [37]	Vegan	CHD Mortality	1	Unclear	HR	0.90	0.60, 1.34	NA	NR Vegetarian Diets	Low
Kaiser et al. 2021 [38]	Vegan	CHD Mortality	2	73,308	Not pooled	Both studies NS	NR	NR	NR	Low
Glenn et al. 2019 [34]	Vegetarian	Stroke Mortality	5	123,638	RR	0.92	0.77, 1.10	44 %	Very Low	Critically Low
Jabri et al. 2021 [36]	Vegetarian	CBVD Mortality	7	124,817	RR	0.84	0.63, 1.14	90 %	Very Low	Critically Low
Jafari et al. 2021 [37]	Vegetarian	CBVD Mortality	5	Unclear	HR	0.93	0.78, 1.10	45 %	NR Vegetarian Diets	Low
Kaiser et al. 2021 [38]	Vegan	CBVD Mortality	1	753	HR	0.70	0.25, 1.98	NA	NR	Low

**Abbreviations:** COE = Certainty of evidence; CHD= coronary heart disease; CVD= cardiovascular disease; HR = Hazard ratio; *N*= number/sample size; NA= not applicable, NR= not reported; NS = Not specified RR = Relative risk; SR = Systematic Review.

1 In Dybvik et al. 2023, mortality was used as a proxy for incidence when incidence was not available.

2 Some studies reported CHD as IHD. These terms were used interchangeably in this umbrella review.

3 Systematic review quality was rated with the AMSTAR2 tool.

4 It was unclear which primary studies were included in the subgroup analysis for vegetarian diets in Wang et al., but it appears authors included the outcome of stroke in CVD incidence.

**Fig. 2:**
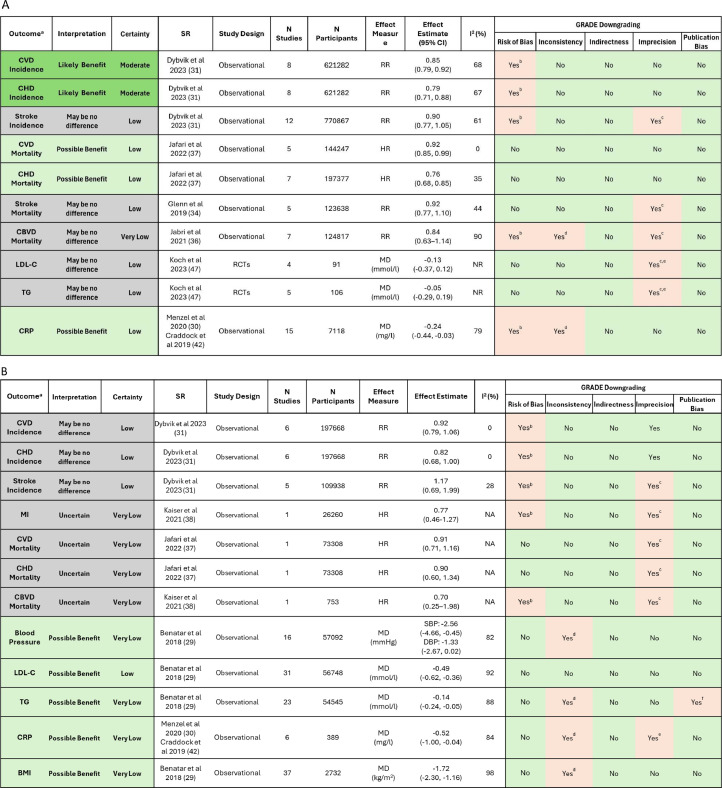
Summary of findings table demonstrating the relationships between (A) Vegetarian and (B) Vegan diets compared to non-vegetarian diets on outcomes of interest.

**Abbreviations:** BMI= body mass index, CBVD= cerebrovascular disease, CHD= coronary heart disease, CI= confidence interval, CRP= *c*-reactive protein, CVD= cardiovascular disease, GRADE= Grading of Recommendation, Assessment, Development, and Evaluation, HR= hazard ratio, LDL-C= low-density lipoprotein cholesterol concentrations, MD= mean difference, MI= myocardial infarction, *N*= number/sample size, NA= not applicable, NR= not reported, RCTs= randomized controlled trials, RR= RR = Relative risk, SR= systematic review, TG= triglyceride concentrations a. The most comprehensive and highest quality SR for each study design was used to assess COE. b. Systematic review authors describe some concerns or high risk of bias in included studies. c. Wide confidence interval and/or confidence interval includes both benefits and harms. d. I^2^>75 % and/or qualitative heterogeneity in results between studies. e. Small or unclear sample sizes. f. Some evidence of publication bias.

Umbrella review authors graded outcomes similarly to Dybvik et al. [31]; COE was downgraded for RoB for vegetarian including vegan dietary patterns, but COE started as ‘high’ because Dybvik et al. [31] utilized the ROBINS-I tool to assess RoB, which is a stricter assessment for observational study designs [49]. Ratings were lower for vegan dietary patterns because the confidence interval included a null effect. COE was moderate for vegetarian and low for vegan dietary patterns (Fig. 2).

### 3.2 Cardiovascular disease events

#### 3.2.1 Stroke

Four SRs examined the relationship between vegetarian dietary patterns and risk of stroke in the target population using cohort studies [31,38,41,44]. One SR focused on interventions but did not identify any primary articles targeting the population of interest [45]. Lu et al. [41] was the highest quality SR followed by Dybvik et al. [31] and Kaiser et al. [38] (Supplementary Table 3). A total of twelve primary observational studies were included in the SRs. All twelve primary studies were reported in Dybvik et al. [31], but only seven were reported in Lu et al. [41].

Diet durations ranged from 5.14 to 28.3 years in Dybvik et al. [31] and 6–26 years in Lu et al. [41] In a meta-analysis of twelve primary studies, there was no impact of vegetarian dietary patterns on risk for total stroke [RR: 0.90 (0.77, 1.05)] (Table 3) [31]. Results were similar in Lu et al. [41], which had higher quality (Supplementary Table 3) and in Quek et al. [44]. In a meta-analysis of five studies, there was no significant relationship between vegan dietary patterns, compared to non-vegetarian dietary patterns, and total stroke [RR: 1.17 (0.69–1.99)]. This effect size was like that found in Kaiser et al. [38] (Table 3). Similarly, there was no significant impact of vegetarian dietary patterns on hemorrhagic or ischemic strokes specifically compared to non-vegetarian dietary patterns [31,38,41,44]. In Dybvik et al. [31], authors described that evidence was rated as “limited-no conclusion” for the impact of vegetarian dietary patterns on reduced risk of total stroke. COE started as high because Dybvik et al. utilized the ROBINS-I tool to assess risk of bias, which is a stricter assessment for observational study designs [31]. In Lu et al. [41], authors described COE as low for vegetarian diet using the Nutrigrade system.

Umbrella review authors agreed with ratings from SR authors, and COE was graded as low using the GRADE method due to RoB and imprecision because the CI included potential for both reduced and increased risk of stroke (Fig. 2). The SR focusing on interventions did not identify any primary articles targeting the population of interest [45].

#### 3.2.2 Myocardial infarction

Myocardial infarction (MI) risk was only reported in Kaiser et al. [38], which included one primary study (EPIC—Oxford). Authors found no impact of vegan dietary patterns, compared to non-vegetarian dietary patterns, on risk of MI [hazard ratio (HR): 0.77 (0.46–1.27)] after up to 18.1 years duration (Table 3) [38].

### 3.3 Cardiovascular disease mortality

SR authors reported associations between vegetarian dietary patterns and total CVD mortality as well as mortality from specific types of CVD, including CHD, CBVD and stroke.

#### 3.3.1 CVD mortality

Four SRs reported the impact of vegetarian including vegan dietary patterns, on CVD mortality [34,37,38,44]. Five primary studies were represented in the four SRs and all these studies were represented in both Glenn et al. [34] and Jafari et al. [37].

The meta-analyses in the SRs demonstrated similar results, though the results in Jafari et al. were statistically significant [HR: 0.92 (0.85, 0.99)] while results in Glenn et al. were not [RR: 0.92 (0.84, 1.02)] (Table 3). Effect sizes were similar in Quek et al. [44] Jafari et al. [37] examined the impact of vegan and lacto-ovo-vegetarian dietary patterns specifically, while Kaiser et al. [38] examined the impact of vegan dietary patterns only. Jafari et al. [37] and Kaiser et al. [38] reported no impact of vegan dietary patterns on CVD mortality risk compared to non-vegetarian dietary patterns.

The effect size for Jafari et al. [37] was used for making conclusions because it was rated as higher quality than Glenn et al. [34] (Supplementary Table 3). Though Glenn et al. [34] reduced COE for imprecision, umbrella review authors did not reduce the grade for this reason, because the quantitative results for vegetarian dietary patterns did not indicate imprecision in Jafari et al. [37] Therefore, COE was rated as low due to observational study designs (Table 3).

### 3.4 CHD mortality

Four SRs examined the relationship between vegetarian or vegan dietary patterns and CHD mortality [34,[36], [37], [38]]. Eight primary studies were represented in the four SRs; Jabri et al. [36] and Jafari et al. [37] each included seven primary studies examining the impact of vegetarian dietary patterns.

Effect sizes were similar and statistically significant in all these SRs. Jafari et al. [37], which was one of the better-quality SRs (Supplementary Table 3), demonstrated that participants following vegetarian including vegan dietary patterns had lower risk of CHD mortality compared to those following non-vegetarian dietary patterns [HR (95 % CI): 0.76 (0.68, 0.85)] (Table 3). The impact of lacto-ovo-vegetarian and vegan dietary patterns specifically were examined in two SRs. Jafari et al. [37] found no significant impact of lacto-ovo-vegetarian or vegan dietary patterns on CHD mortality compared to non-vegetarian dietary patterns (Table 3). Kaiser et al. [38] found no significant relationship between vegan, compared to omnivorous, dietary patterns and CHD in two primary studies. Jabri et al. [36], Jafari et al. [37] and Glenn et al. [34] each downgraded evidence for observational study designs. Glenn additionally downgraded COE for indirectness as some included cohorts were health-conscious groups that may not represent the general population.

For vegetarian dietary patterns, umbrella review authors chose to mark COE down for observational evidence only, because the predominant health-conscious behavior of concern is, in this case, the behavior of interest, and COE was low. For vegan dietary patterns, umbrella review authors utilized findings from the higher quality SR [37] and rated COE as very low due to observational study designs and a CI ranging from a reduced risk to increased risk (Fig. 2).

### 3.5 Stroke mortality

One SR examined the impact of vegetarian dietary patterns on stroke mortality [34]. Glenn et al. [34] included five primary studies in meta-analysis and found no significant impact of vegetarian including vegan dietary patterns on risk of stroke mortality [RR: 0.92 (0.77, 1.10)] (Table 3). Using the GRADE method, Glenn et al. [34] rated COE as very low due to indirectness, because participants consuming vegetarian dietary patterns were part of health-conscious groups, and imprecision.

Umbrella review authors did not agree that evidence should be marked down for indirectness but did agree evidence should be marked down for imprecision because the CI is wide and includes both potential reduction and increase in risk (Fig. 2).

### 3.6 Cerebrovascular disease (CBVD) mortality

Three SRs examined the relationship between vegetarian dietary patterns and CBVD mortality in the target population [[36], [37], [38]]. In the two SRs examining the impact of vegetarian dietary patterns [36,37], eight primary studies were represented, and five of these were included in both SRs. Neither SR demonstrated a significant impact of vegetarian including vegan dietary patterns on risk of CBVD mortality (Table 3). While Jafari et al. [37] had higher quality (Supplementary Table 3), Jabri et al. [36] was more comprehensive and the effect size was RR (95 % CI): 0.84 (0.63–1.14). Kaiser et al. [38] found no significant relationship between vegan, compared to omnivorous, dietary patterns and risk of mortality from CBVD (Table 3). Jabri et al. [36] described COE as very low due to RoB, inconsistency (I^2^=90 %), and publication bias. It was unclear why Jabri et al. [36] rated down for publication bias because they describe that they do not assess publication bias for analyses with <10 studies, and no data on publication bias was provided. Jafari et al. [37] rated evidence for CBVD as very low due to observational study design and imprecision (CI includes potential for both decreased and increased risk).

Umbrella review author described evidence certainty was very low for vegetarian dietary patterns due to RoB, inconsistency between studies and a wide confidence interval for the effect size. The impact of vegan dietary patterns on CBVD mortality was also very low (Fig. 2).

### 3.7 Hypertension incidence and overweight/obesity incidence

No included SRs reported on the relationship between vegetarian dietary patterns, compared to non-vegetarian dietary patterns, and risk of hypertension or overweight/obesity in presumably healthy adults in the general population.

### 3.8 Blood pressure

Seven SRs examined the relationship between vegetarian including vegan dietary patterns and BP. One SR examined evidence from observational studies [29], and six SRs examined results from controlled trials [33,39,40,43,45,46].

Benatar et al. [29] examined the relationship between following vegan diets for at least one year and BP in 16 primary observational studies. In meta-analysis, vegan diets were associated with lower systolic BP [MD (95 % CI): −2.56 mmHg (−4.66, −0.45); I^2^=83 %], but results were not significant for diastolic BP [−1.33 mmHg (−2.67, 0.02); I^2^=82 %]. The relationship was largely driven by participants from non-Eastern Asian countries. The six SRs that utilized controlled trials of vegetarian or vegan dietary patterns [33,39,40,43,45,46] collectively included 24 primary studies after de-duplication. However, 19 of these studies targeted adults with diagnosed diseases such as type 2 diabetes, hypertension, overweight or obesity, and/or heart disease, and SR conclusions were not considered applicable to the general population.

COE was very low when assessed by umbrella review authors due to observational study design and high heterogeneity when comparing results between studies (Fig. 2).

### 3.9 LDL-Cholesterol & triglyceride concentrations

Seven SRs examined the relationship between vegetarian, including vegan, dietary patterns and LDL-cholesterol and/or triglyceride concentrations [29,32,35,43,[45], [46], [47]]. Six SRs examined evidence from controlled trials [32,35,43,[45], [46], [47]], but available evidence was not applicable to disease prevention in the general population in five of these SRs [32,35,43,45,46]. One SR of RCTs included sub-group analysis to examine effects of vegetarian dietary patterns in presumably healthy adults [47]. The SR that examined evidence from observational studies required adults to have no significant co-morbidities and follow a vegan diet for at least one year [29].

The SR with RCTs described there may be no effect of vegetarian and vegan dietary patterns combined on LDL cholesterol concentrations in four RCTs in presumably healthy adults [−0.13 mmol/l (−0.37, 0.12)] [47]. In addition, there may be no effect of vegetarian and vegan dietary patterns combined on triglyceride concentrations in five RCTs in presumably healthy adults [−0.05 mmol/l (−0.29, 0.10) [47].

In the SR and meta-analysis of 31 observational studies, vegan dietary patterns may be associated with significantly lower LDL-cholesterol concentrations of −0.49 mmol/l (−0.62, −0.36) (*p* < 0.0001) compared to omnivorous diets, though heterogeneity was high (I^2^=92 %) [29]. In the SR and meta-analysis of 29 observational studies, vegan dietary patterns may be associated with significantly lower triglyceride concentrations of −0.05 mmol/l (−0.24, −0.05) (*p* = 0.004) compared to omnivorous diets, and heterogeneity was high [29]. Most included primary studies examining LDL cholesterol and triglyceride concentrations were rated as “high quality” using the Newcastle-Ottawa Scale.

For the impact of vegetarian dietary patterns on LDL-cholesterol, COE was graded as low due to very small sample sizes and a wide CI that included potential for both increased and decreased LDL-cholesterol concentrations (Fig. 2). When grading evidence on the impact of vegan dietary patterns and LDL cholesterol concentrations, we chose not to reduce COE for imprecision, because nearly all studies demonstrated a benefit of vegan dietary patterns, and COE was low. For the impact of vegetarian including vegan dietary patterns on triglyceride concentrations, COE was low due to very small sample sizes and a wide confidence interval that includes potential for both increased and decreased triglyceride concentrations (Fig. 2). For the impact of vegan dietary patterns on triglyceride concentrations, COE was very low when assessed by umbrella review authors due to observational study designs, inconsistency between studies and publication bias (Fig. 2).

### 3.10 CRP concentrations

Three SRs examined the relationship between vegetarian dietary patterns and CRP concentrations in presumably healthy adults in the general population [30,32,42]. Two SRs examined evidence from observational studies [30,42], and two SRs examined evidence from controlled trials [30,32]. Evidence from controlled trials did not address the population of interest and umbrella review conclusions were primarily based on observational evidence, which assessed large, population-based studies. In the two SRs utilizing observational (cross-sectional) studies, there were 22 primary studies represented in the SRs after de-duplication, and 13 of these studies were reported in both SRs [30,42].

Data from both SR meta-analyses were combined in a novel meta-analysis conducted by umbrella review authors. Meta-analysis of two SRs including 22 primary studies with 8250 participants, demonstrated a statistically significant lower CRP concentration in all vegetarian and vegan dietary patterns combined compared with non-vegetarian dietary patterns, and heterogeneity was very high [MD (95 % CI): −0.55 mg/l (−1.07, −0.03); I^2^=99.5 %] (Supplementary Fig. 2a–c). Two SRs examined the effect of vegetarian including vegan diets using intervention studies [30,32]. Quantitative results for non-specified vegetarian dietary patterns did not reach statistical significance, likely because only three studies were included and heterogeneity with was very high (I^2^=99.9 %) (Supplementary Fig. 2a). Studies with low risk of bias demonstrated a significant effect, while those with some risk of bias demonstrated did not (Supplementary Fig. 2b). Publication bias was not detected (Supplementary Fig. 2c).

Umbrella review authors graded COE as very low due to observational study designs, RoB and inconsistency between studies, which aligned with evidence grading in the SRs (Fig. 2) [30,42].

### 3.11 Body mass index (BMI)

Two SRs examined the relationship between vegan dietary patterns, compared to non-vegetarian dietary patterns, and BMI in the target population using observational studies [28,29]. Four SRs examined this relationship using intervention studies [35,43,45,46]. In the four SRs of intervention studies combined, 13 of the 14 primary studies represented specifically targeted adults with disease or high disease risk and results were not applicable to the target population. In both SRs with observational studies combined, 64 primary observational studies were represented, and there was an overlap of six primary studies between SRs [28,29]. Benatar et al. [29] demonstrated vegans had a lower BMI than omnivores [−1.72 kg/m^2^ (−2.52, −1.32); I^2^=98 %]. Bakaloudi et al. [28] did not include quantitative results or assess COE, but reported similar direction of findings and RoB assessment as Benatar et al. [29].

Umbrella review authors graded COE as very low due to observational study designs and high heterogeneity between studies (Fig. 2).

## 4 Discussion

This umbrella review of published SRs and meta-analyses examined the relationships between vegetarian including vegan dietary patterns, compared to non-vegetarian dietary patterns, and prevention of cardiovascular risk factors, disease and mortality. Consumption of vegetarian including vegan dietary patterns was associated with reduced risk for CVD and CHD incidence and mortality compared to non-vegetarian dietary patterns. Vegan dietary patterns were associated with mitigated CVD risk factors such as lower BP, LDL-C, triglycerides, CRP, and BMI when compared to non-vegetarian dietary patterns. Effect sizes and direction of results were generally consistent between all included SRs for the outcomes of CVD incidence, events and mortality (Table 3). SRs with the target population were more sparse for intermediate outcomes, but were generally consistent in reporting improvement from vegan dietary patterns. An overview of the umbrella review and results can be found in Central Illustration. These findings can be used in the development and refinement of clinical guidelines for CVD prevention.

Across most risk factor and disease outcomes reviewed, there was generally low COE due to inherent limitations in examining dietary patterns for disease prevention in free-living adults, including reliance on observational study designs and their associated biases. In addition, biomarker outcomes had high heterogeneity in results. Though the source of this heterogeneity is not elucidated in the included SRs, it may result from differences in the quality of vegetarian and comparison dietary patterns, time following the dietary patterns, or variability in cardiometabolic risk factors in participants in the general population. In the outcomes reviewed, only lower CVD and CHD incidence had moderate certainty evidence for following vegetarian compared to non-vegetarian dietary patterns. Findings in this umbrella review were consistent with those reported in recent SRs and meta-analysis examining similar research questions [50,51]. Despite minor differences in population or outcome parameters compared to this review, results across reviews consistently suggest that consuming vegetarian including vegan dietary patterns are associated with improvements in several important cardiovascular risk factors.

### 4.1 Practice implications

There is robust evidence supporting vegetarian and vegan dietary patterns to promote cardiometabolic health [9,10,13]. Individuals may choose to adopt a vegetarian-style dietary pattern for a variety of reasons, including ethical concerns for the treatment of animals and protection of the environment, mitigation of green-house gas emissions, and to therapeutically manage or lower the risk of several chronic diseases [52,53]. Similar to other dietary patterns, vegetarian dietary patterns can be followed in healthy and less healthy ways [14]. Following a vegetarian-style dietary pattern that mainly consists of low-quality foods and beverages (such as those high in refined carbohydrates, added sugars, and saturated fat) could be unhealthy and increase the risk of CVD when compared to non-vegetarian dietary patterns [14,54,55].

**Fig. 3:**
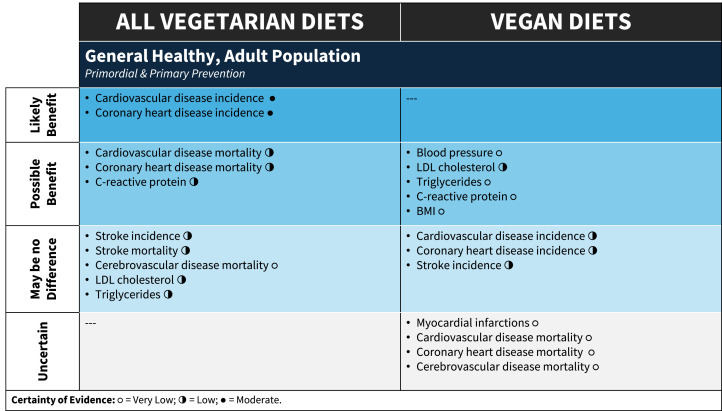
Evidence on the impact of vegetarian dietary patterns for the general population to aid in shared decision-making with clients.

**Abbreviations:** BMI= body mass index; LDL= low-density lipoprotein Certainty of Evidence: °= Very Low; ◑ = Low; ● = Moderate.

Nutrition misinformation [56], misplaced emphasis on a food or dietary component or the lack of nuance and oversimplification of dietary approaches may lead to adoption of dietary patterns that do not optimally support health [3]. The diet quality of vegetarian compared with non-vegetarian dietary patterns can be dependent on the index or measure used to assess diet quality. However, it is more greatly impacted by the foods vegetarians consumed in place of meat, fish, and poultry in their diets (e.g., replacing animal-based protein with refined carbohydrates) [57]. A recent scientific statement from the American Heart Association found that vegetarian dietary patterns (including ovo, lacto, and ovo/lacto) were in the top tier of popular dietary patterns that were in alignment with the organization's dietary guidance (along with the Mediterranean, DASH [Dietary Approaches to Stop Hypertension] and pescetarian diets) [58]. *A priori*, we decided to look for impact of healthfulness of vegetarian dietary patterns, but there was little/no information available in the SRs. This should be a focus for future analyses as previous research has found that a higher quality plant-based dietary pattern compared to a lower quality plant-based diet was associated with improvements in obesity, mortality, diabetes, and cardiovascular disease [57]. Practitioners can guide clients in discerning between healthy and unhealthy components of dietary patterns by focusing nutrition education and counseling on well-established features of heart-healthy vegetarian and vegan dietary patterns (e.g., vegetables, fruits, legumes, whole grains, and incorporation of healthy sources of unsaturated fats). Fig. 3 may be used to transparently guide shared decision making with clients or patients regarding if a vegetarian or vegan dietary patterns can achieve health goals.

**Central Illustration:**
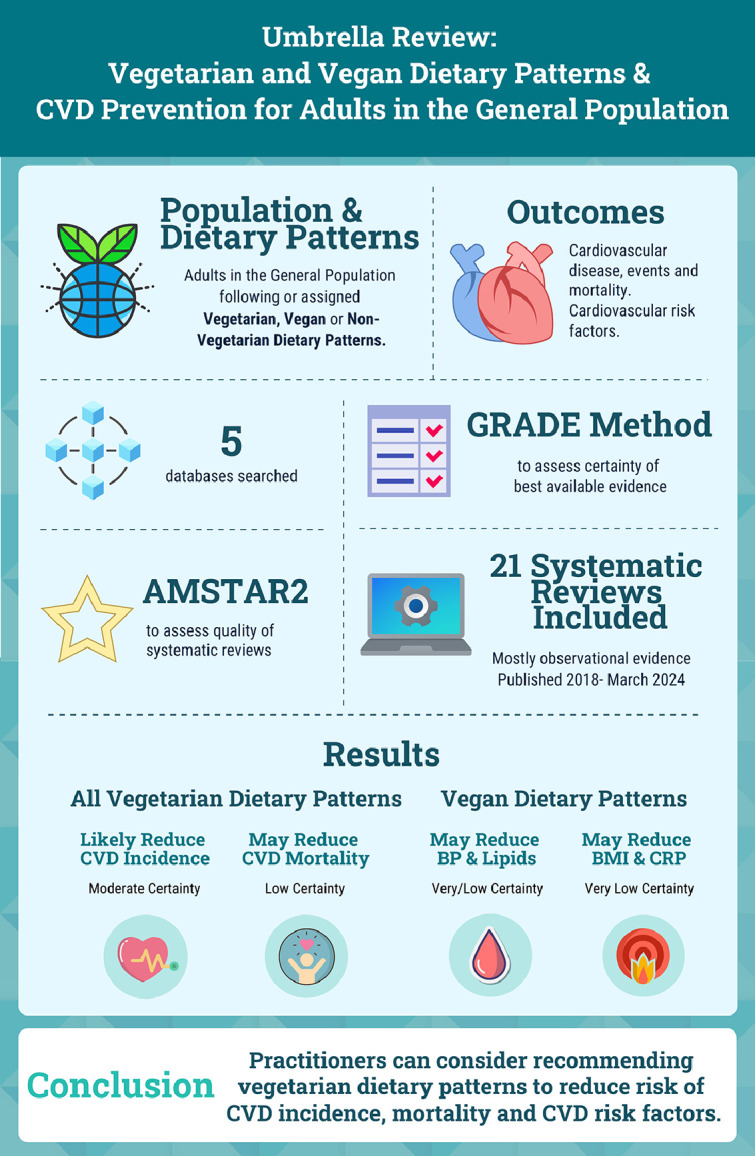
Summary of umbrella review examining the impact of vegetarian dietary patterns on cardiovascular risk factors and disease prevention for the general population.

**Abbreviations:** AMSTAR2= *A* Measurement Tool to Assess Systematic Reviews, version 2; BMI= body mass index; BP= blood pressure; CRP= *c*-reactive protein; CVD= cardiovascular disease; GRADE= Grading of Recommendations, Assessment, Development, and Evaluation

### 4.2 Strengths and limitations

A strength of our umbrella review was the broad and inclusive approach based on a prespecified research protocol, rigorous search using five databases, and using a well-established quality assessment tool. Several limitations of our review warrant discussion. A key challenge when interpreting studies included in this review was the lack of a clear definition of vegetarian and vegan dietary patterns [6]. This was further complicated as some studies reported associations or RRs for vegetarian or vegan dietary patterns in isolation, while others combined the two patterns. Additionally, within observational studies, participants may have consumed meat/poultry/fish ≤once/month but were still classified as “vegetarian”. Overall, categorization of dietary patterns as “vegetarian” or “vegan” was inconsistent and variably defined by primary study authors. Also, we were not able to assess dietary adherence and nutritional quality of dietary patterns as this was not reported within SRs. We acknowledge these are impactful variables that should be reported and considered when interpreting results of nutrition trials [59]. Third, there were gaps in available SRs characterizing the health impacts of vegetarian and vegan dietary patterns on the outcomes MI, hypertension incidence, overweight and obesity incidence, and BMI. Additionally, the included SRs utilized different tools for assessing risk of bias of primary studies and assessments were conducted by many different researchers, which may lead to variation in risk of bias assessments.

Finally, lack of SRs with RCTs targeting CVD prevention for generally healthy adults led to a reliance on SRs of observational studies. However, observational studies, particularly long-term prospective cohort studies, can be an invaluable resource in answering nutritional questions in regard to long-term health [60]. A forthcoming manuscript from our group will discuss the impact of vegetarian dietary patterns on adults with chronic diseases (e.g., type 2 diabetes).

## 5 Conclusion

This umbrella review of SRs found that in presumably healthy adults in the general population, vegetarian and vegan dietary patterns were associated with lower CVD and CHD incidence, and risk of CVD mortality compared to non-vegetarian dietary patterns. Vegan dietary patterns were associated with lower triglyceride, LDL-C, and CRP concentrations, BP, and BMI. Across most outcomes reviewed, there was generally low COE due to reliance on observational study designs and high heterogeneity between studies. Therefore, additional high-quality RCTs examining the effects of vegetarian dietary patterns compared to non-vegetarian dietary patterns for CVD prevention among generally healthy adults are needed. Clinicians may consider recommending vegetarian dietary patterns to reduce cardiometabolic risk factors and decrease the risk of CVD incidence and mortality.

## Role of the funder/sponsor

The funder had no role in the design and conduct of the study; collection, analysis and interpretation of the data; preparation, review, or approval of manuscript; and decision to submit the manuscript for publication.

## Data sharing statement

Data described in the manuscript and analytic code will be made available upon request pending application and approval from manuscript authors.

## Funding support

This systematic review was supported by the Academy of Nutrition and Dietetics, the Academy of Nutrition and Dietetics Foundation, and the Academy of Nutrition and Dietetics Vegetarian Nutrition Dietetic Practice Group [no grant number].

## CRediT authorship contribution statement

**Matthew J. Landry:** Writing – review & editing, Writing – original draft, Visualization, Conceptualization. **Katelyn E. Senkus:** Project administration, Methodology, Formal analysis, Data curation, Conceptualization. **A Reed Mangels:** Writing – review & editing, Conceptualization. **Nanci S. Guest:** Writing – review & editing, Conceptualization. **Roman Pawlak:** Writing – review & editing, Conceptualization. **Sudha Raj:** Writing – review & editing, Conceptualization. **Deepa Handu:** Writing – review & editing, Supervision, Methodology, Conceptualization. **Mary Rozga:** Writing – review & editing, Writing – original draft, Visualization, Supervision, Project administration, Methodology, Formal analysis, Data curation, Conceptualization.

## Declaration of competing interest

The authors declare the following financial interests/personal relationships which may be considered as potential competing interests: Mary Rozga reports financial support was provided by Academy of Nutrition and Dietetics. Mary Rozga reports financial support was provided by Academy of Nutrition and Dietetics Foundation. Mary Rozga reports financial support was provided by Academy of Nutrition and Dietetics Vegetarian Nutrition Dietetic Practice Group. If there are other authors, they declare that they have no known competing financial interests or personal relationships that could have appeared to influence the work reported in this paper.

## Appendix Supplementary materials

Image, application 1

## References

[1] Lichtenstein A.H., Appel L.J., Vadiveloo M., Hu F.B., Kris-Etherton P.M., Rebholz C.M. (2021). 2021 dietary guidance to improve cardiovascular health: a scientific statement from the American heart association. Circulation.

[2] Virani S.S., Newby L.K., Arnold S.V., Bittner V., Brewer L.C., Demeter S.H. (2023). 2023 AHA/ACC/ACCP/ASPC/NLA/PCNA guideline for the management of patients with chronic coronary disease: a report of the American Heart Association/American College of Cardiology Joint Committee on Clinical Practice Guidelines. Circulation.

[3] Gardner C.D., Vadiveloo M.K., Petersen K.S., Anderson C.A., Springfield S., Van Horn L. (2023). Popular dietary patterns: alignment with American Heart Association 2021 dietary guidance: a scientific statement from the American Heart Association. Circulation.

[4] Cara K.C., Goldman D.M., Kollman B.K., Amato S.S., Tull M.D., Karlsen M.C. (2023). Commonalities among dietary recommendations from 2010 to 2021 clinical practice guidelines: a meta-epidemiological study from the American College of Lifestyle Medicine. Adv Nutr.

[5] Landry M.J., Ward C.P. (2024). Health benefits of a plant-based dietary pattern and implementation in healthcare and clinical practice. Am J Lifestyle Med.

[6] Storz M.A. (2022). What makes a plant-based diet? A review of current concepts and proposal for a standardized plant-based dietary intervention checklist. Eur J Clin Nutr.

[7] Melina V., Craig W., Levin S. (2016). Position of the academy of nutrition and dietetics: vegetarian diets. J Acad Nutr Diet.

[8] Hargreaves S.M., Rosenfeld D.L., Moreira A.V.B., Zandonadi R.P. (2023). Plant-based and vegetarian diets: an overview and definition of these dietary patterns. Eur J Nutr.

[9] Satija A., Hu F.B. (2018). Plant-based diets and cardiovascular health. Trends Cardiovasc Med.

[10] Kahleova H., Levin S., Barnard N.D (2018). Vegetarian dietary patterns and cardiovascular disease. Prog Cardiovasc Dis.

[11] Glenn A.J., Guasch-Ferré M., Malik V.S., Kendall C.W., Manson J.E., Rimm E.B. (2023). Portfolio diet score and risk of cardiovascular disease: findings from 3 prospective cohort studies. Circulation.

[12] Neuenschwander M., Stadelmaier J., Eble J., Grummich K., Szczerba E., Kiesswetter E. (2023). Substitution of animal-based with plant-based foods on cardiometabolic health and all-cause mortality: a systematic review and meta-analysis of prospective studies. BMC Med.

[13] Oussalah A., Levy J., Berthezène C., Alpers D.H., Guéant J.L. (2020). Health outcomes associated with vegetarian diets: an umbrella review of systematic reviews and meta-analyses. Clin Nutr.

[14] Satija A., Bhupathiraju S.N., Spiegelman D., Chiuve S.E., Manson J.E., Willett W. (2017). Healthful and unhealthful plant-based diets and the risk of coronary heart disease in US adults. J Am Coll Cardiol.

[15] Handu D., Piemonte T. (2022). Dietary approaches and health outcomes: an evidence analysis center scoping review. J Acad Nutr Diet.

[16] Higgins J.P.T., Thomas J., Chandler J., Cumpston M., Li T., Page M.J., Welch V.A. (2022). Cochrane handbook for systematic reviews of interventions version 6.3 cochrane.

[17] Page M.J., McKenzie J.E., Bossuyt P.M., Boutron I., Hoffmann T.C., Mulrow C.D. (2021). The PRISMA 2020 statement: an updated guideline for reporting systematic reviews. BMJ.

[18] Rozga M., Senkus K., Raj S., Mangels R., Landry M, Guest N., Pawlak R., Craig W. Impact of Vegetarian, Vegan and Non-Vegetarian Diets on Cardiometabolic Disease Prevention, Secondary Prevention and Management in Adults. PROSPERO 2023 CRD42023396453 Available from: https://www.crd.york.ac.uk/prospero/display_record.php?ID=CRD42023396453.

[19] Academy of Nutrition and Dietetics Evidence Analysis Library. “In adults with CVD risk factors (e.g., overweight/obesity, dyslipidemia, hypertension), CVD or T2DM, what are the effects of vegetarian diets on disease-specific outcomes of interest, including CVD risk factors, type 2 diabetes mellitus, and cardiovascular disease.” Accessed 30 September 2024: https://www.andeal.org/topic.cfm?menu=5271.

[20] Ouzzani M., Hammady H., Fedorowicz Z., Elmagarmid A. (2016). Rayyan-a web and mobile app for systematic reviews. Syst Rev.

[21] The AMSTAR Team. AMSTAR 2 – the new and improved AMSTAR 2021. Bruyère Research Institute. Ontario, Canada [Available from: https://amstar.ca/Amstar-2.php.]. Accessed September 30, 2024.

[22] Shea B.J., Reeves B.C., Wells G., Thuku M., Hamel C., Moran J. (2017). AMSTAR 2: a critical appraisal tool for systematic reviews that include randomised or non-randomised studies of healthcare interventions, or both. BMJ.

[23] Dettori J.R., Norvell D.C., Chapman J.R. (2022). Fixed-effect vs random-effects models for meta-analysis: 3 points to consider. Glob Spine J.

[24] Wallace B.C., Dahabreh I.J., Trikalinos T.A., Lau J., Trow P., Schmid C.H. (2012). Closing the gap between methodologists and end-users: R as a computational back-end. J Stat Softw.

[25] RStudio Team (2019). RStudio: Integrated Development for R. RStudio, Inc., Boston, MA. (version 1.2.5029). URL http://www.rstudio.com/.

[26] Guyatt G., Oxman A.D., Sultan S., Brozek J., Glasziou P., Alonso-Coello P. (2013). GRADE guidelines: 11. Making an overall rating of confidence in effect estimates for a single outcome and for all outcomes. J Clin Epidemiol.

[27] GRADEpro GDT: GRADEpro Guideline Development Tool [Software]. McMaster University and Evidence Prime, 2024. Available from https://www.gradepro.org/.

[28] Bakaloudi D.R., Halloran A., Rippin H.L., Oikonomidou A.C., Dardavesis T.I., Williams J. (2021). Intake and adequacy of the vegan diet. A systematic review of the evidence. Clin Nutr.

[29] Benatar J.R., Stewart R.A.H. (2018). Cardiometabolic risk factors in vegans; A meta-analysis of observational studies. PLoS One.

[30] Craddock J.C., Neale E.P., Peoples G.E., Probst Y.C. (2019). Vegetarian-based dietary patterns and their relation with inflammatory and immune biomarkers: a systematic review and meta-analysis. Adv Nutr.

[31] Dybvik J.S., Svendsen M., Aune D. (2023). Vegetarian and vegan diets and the risk of cardiovascular disease, ischemic heart disease and stroke: a systematic review and meta-analysis of prospective cohort studies. Eur J Nutr.

[32] Elliott P.S., Kharaty S.S., Phillips C.M. (2022). Plant-based diets and lipid, lipoprotein, and inflammatory biomarkers of cardiovascular disease: a review of observational and interventional studies. Nutrients.

[33] Gibbs J., Gaskin E., Ji C., Miller M.A., Cappuccio F.P (2021). The effect of plant-based dietary patterns on blood pressure: a systematic review and meta-analysis of controlled intervention trials. J Hypertens.

[34] Glenn A.J., Viguiliouk E., Seider M., Boucher B.A., Khan T.A., Blanco Mejia S. (2019). Relation of vegetarian dietary patterns with major cardiovascular outcomes: a systematic review and meta-analysis of prospective cohort studies. Front Nutr.

[35] Ivanova S., Delattre C., Karcheva-Bahchevanska D., Benbasat N., Nalbantova V., Ivanov K (2021). Plant-based diet as a strategy for weight control. Foods.

[36] Jabri A., Kumar A., Verghese E., Alameh A., Kumar A., Khan M.S. (2021). Meta-analysis of effect of vegetarian diet on ischemic heart disease and all-cause mortality. Am J Prev Cardiol.

[37] Jafari S., Hezaveh E., Jalilpiran Y., Jayedi A., Wong A., Safaiyan A. (2022). Plant-based diets and risk of disease mortality: a systematic review and meta-analysis of cohort studies. Crit Rev Food Sci Nutr.

[38] Kaiser J., van Daalen K.R., Thayyil A., Cocco M., Caputo D., Oliver-Williams C. (2021). A systematic review of the association between vegan diets and risk of cardiovascular disease. J Nutr.

[39] Lee K.W., Loh H.C., Ching S.M., Devaraj N.K., Hoo F.K. (2020). Effects of vegetarian diets on blood pressure lowering: a systematic review with meta-analysis and trial sequential analysis. Nutrients.

[40] Lopez P.D., Cativo E.H., Atlas S.A., Rosendorff C. (2019). The effect of vegan diets on blood pressure in adults: a meta-analysis of randomized controlled trials. Am J Med.

[41] Lu J.W., Yu L.H., Tu Y.K., Cheng H.Y., Chen L.Y., Loh C.H. (2021). Risk of incident stroke among vegetarians compared to nonvegetarians: a systematic review and meta-analysis of prospective cohort studies. Nutrients.

[42] Menzel J., Jabakhanji A., Biemann R., Mai K., Abraham K., Weikert C (2020). Systematic review and meta-analysis of the associations of vegan and vegetarian diets with inflammatory biomarkers. Sci Rep.

[43] Pollakova D., Andreadi A., Pacifici F., Della-Morte D., Lauro D., Tubili C. (2021). The impact of vegan diet in the prevention and treatment of type 2 diabetes: a systematic review. Nutrients.

[44] Quek J., Lim G., Lim W.H., Ng C.H., So W.Z., Toh J. (2021). The Association of plant-based diet with cardiovascular disease and mortality: a meta-analysis and systematic review of prospect cohort studies. Front Cardiovasc Med.

[45] Rees K., Al-Khudairy L., Takeda A., Stranges S (2021). Vegan dietary pattern for the primary and secondary prevention of cardiovascular diseases. Cochrane Database Syst Rev.

[46] Remde A., DeTurk S.N., Almardini A., Steiner L., Wojda T. (2022). Plant-predominant eating patterns - how effective are they for treating obesity and related cardiometabolic health outcomes? - a systematic review. Nutr Rev.

[47] Koch C.A., Kjeldsen E.W., Frikke-Schmidt R. (2023). Vegetarian or vegan diets and blood lipids: a meta-analysis of randomized trials. Eur Heart J.

[48] Wang Y., Liu B., Han H., Hu Y., Zhu L., Rimm E.B. (2023). Associations between plant-based dietary patterns and risks of type 2 diabetes, cardiovascular disease, cancer, and mortality - a systematic review and meta-analysis. Nutr J.

[49] Schünemann H.J., Cuello C., Akl E.A., Mustafa R.A., Meerpohl J.J., Thayer K. (2019). GRADE guidelines: 18. How ROBINS-I and other tools to assess risk of bias in nonrandomized studies should be used to rate the certainty of a body of evidence. J Clin Epidemiol.

[50] Wang T., Kroeger C.M., Cassidy S., Mitra S., Ribeiro R.V., Jose S. (2023). Vegetarian dietary patterns and cardiometabolic risk in people with or at high risk of cardiovascular disease: a systematic review and meta-analysis. JAMA Netw Open.

[51] Ocagli H., Berti G., Rango D., Norbiato F., Chiaruttini M.V., Lorenzoni G. (2023). Association of vegetarian and vegan diets with cardiovascular health: an umbrella review of meta-analysis of observational studies and randomized trials. Nutrients.

[52] Miki A.J., Livingston K.A., Karlsen M.C., Folta S.C., McKeown N.M. (2020). Using evidence mapping to examine motivations for following plant-based diets. Curr Dev Nutr.

[53] Mendoza-Vasconez A.S., Landry M.J., Crimarco A., Bladier C., Gardner C.D. (2021). Sustainable diets for cardiovascular disease prevention and management. Curr Atheroscler Rep.

[54] Thompson A.S., Tresserra-Rimbau A., Karavasiloglou N., Jennings A., Cantwell M., Hill C. (2023). Association of healthful plant-based diet adherence with risk of mortality and major chronic diseases among adults in the UK. JAMA Netw Open.

[55] Hemler E.C., Hu F.B. (2019). Plant-based diets for cardiovascular disease prevention: all plant foods are not created equal. Curr Atheroscler Rep.

[56] Diekman C., Ryan C.D., Oliver T.L. (2023). Misinformation and disinformation in food science and nutrition: impact on practice. J Nutr.

[57] Parker H.W., Vadiveloo M.K. (2019). Diet quality of vegetarian diets compared with nonvegetarian diets: a systematic review. Nutr Rev.

[58] Gardner C.D., Vadiveloo M.K., Petersen K.S., Anderson C.A.M., Springfield S., Van Horn L. (2023). Popular dietary patterns: alignment With American Heart Association 2021 dietary guidance: a scientific statement from the American Heart Association. Circulation.

[59] Gardner C.D., Crimarco A., Landry M.J., Fielding-Singh P. (2020). Nutrition study design issues—important issues for interpretation. Am J Health Promot.

[60] Satija A., Stampfer M.J., Rimm E.B., Willett W., Hu F.B. (2018). Perspective: are large, simple trials the solution for nutrition research?. Adv Nutr.

