# Supplementary material for: Vegetarian dietary patterns and cardiovascular risk factors and disease prevention: An umbrella review of systematic reviews
Source: Am J Prev Cardiol. 2024 Sep 28;20:100868. doi: 10.1016/j.ajpc.2024.100868 (PMC11489049; doi:10.1016/j.ajpc.2024.100868)
Supplement: Supplementary file 1 [file mmc1.docx]

**Supplementary Information for**

**Vegetarian Dietary Patterns and Cardiovascular Risk Factors and Disease Prevention:**

**An Umbrella Review of Systematic Reviews**

- **Supplementary Figure 1.** Variety and description of vegetarian dietary patterns.
- **Supplementary Figure 2**. Forest Plot of Meta-analyses from an Umbrella Review Including Two Systematic Reviews Examining the Relationship between Vegetarian Diets and C-reactive Protein (mg/l), Including Sub-Group Analyses for A) Vegetarian Diet Type and B) Study Quality and C) Funnel Plot for Publication Bias
- **Supplementary Table 1.** Search Plan for Umbrella Review Examining the Relationships between Vegetarian, Vegan and Non-Vegetarian Diets
- **Supplementary Table 2.** List of Systematic Reviews Excluded in the Umbrella Review Examining the Research Question: In presumably healthy adults in the general population, what is the relationship between vegetarian diets, compared to non-vegetarian diets, on outcomes of interest?
- **Supplementary Table 3**. AMSTAR2 Ratings in Critical Systematic Review Domains and Overall Certainty in Results for Systematic Reviews Included in the Umbrella Review Examining the Relationships Between a Vegetarian/Vegan Diet, Compared to a Non-Vegetarian Diet and Cardiovascular Risk and Disease Outcomes

**Supplementary Figure 1.**  Variety and description of vegetarian dietary patterns^a^


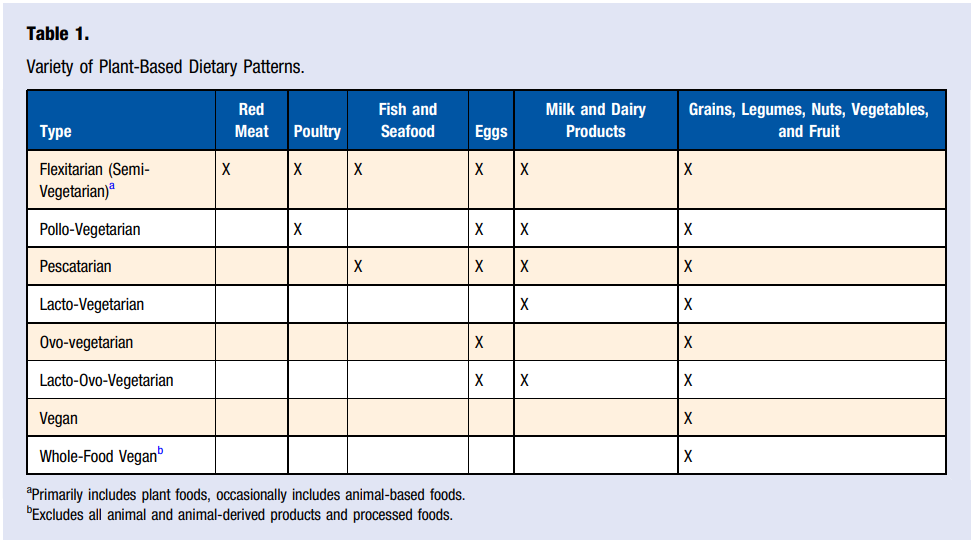


^a^Reprinted with permission from Landry et al 2024 ^1^

**Supplementary** **Figure 2.** Forest Plot of Meta-analyses from an Umbrella Review Including Two Systematic Reviews Examining the Relationship between Vegetarian Diets and C-reactive Protein (mg/l), Including Sub-Group Analyses for A) Vegetarian Diet Type and B) Study Quality and C) Funnel Plot for Publication Bias

A


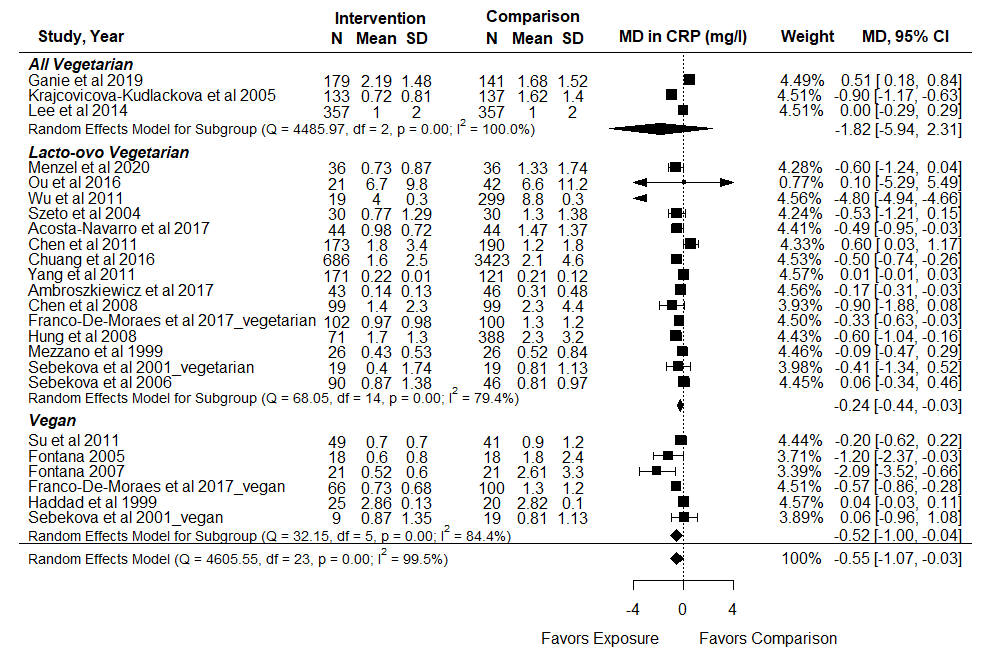


B


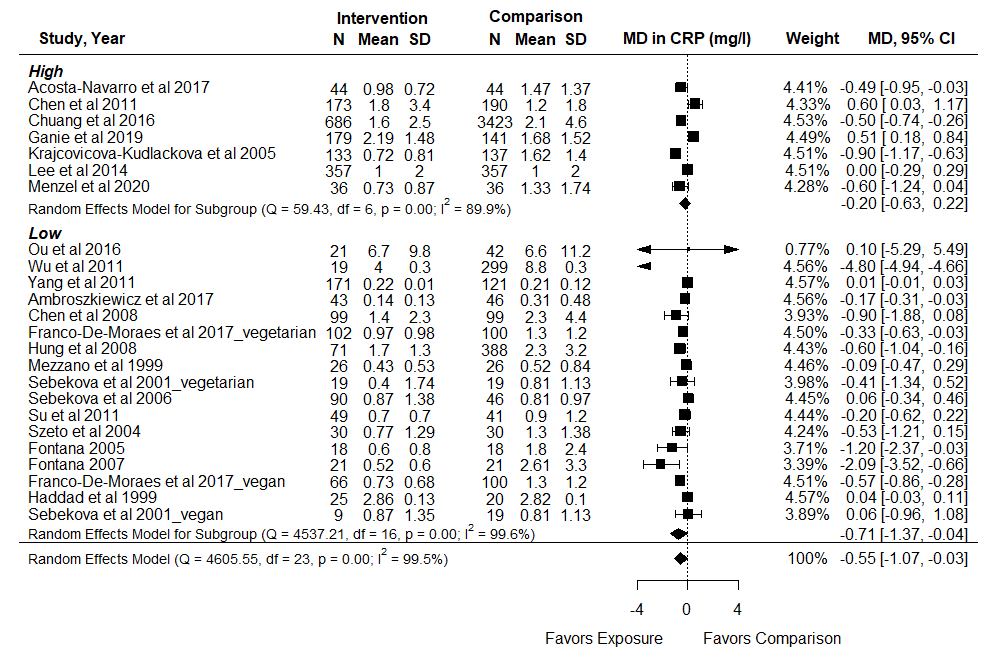


C


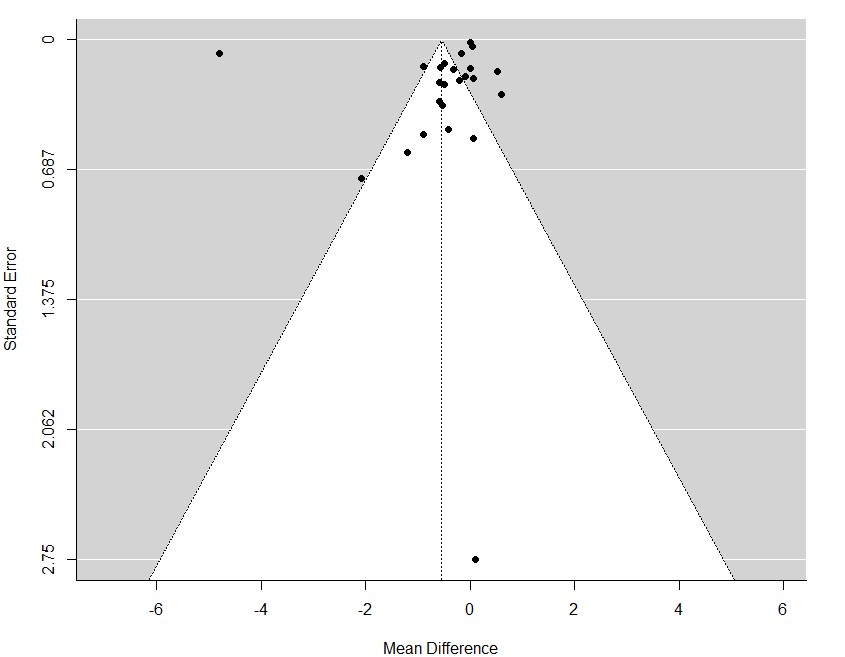


**Supplementary Table 1. Search Plan for Umbrella Review Examining the Relationships between Vegetarian, Vegan and Non-Vegetarian Diets**

| **MEDLINE (Ebsco)** | | | |
| --- | --- | --- | --- |
| **#** | **Query** | **Limiters/Expanders** | **Last Run Via** |
| S9 | S8 AND DT 20180101-20240101 | Search modes - Boolean/Phrase | Interface - EBSCOhost Research Databases  Search Screen - Advanced Search  Database - MEDLINE Complete |
| S8 | S7 AND LA English | Search modes - Boolean/Phrase | Interface - EBSCOhost Research Databases  Search Screen - Advanced Search  Database - MEDLINE Complete |
| S7 | S6 NOT ((MH "Animals") NOT ((MH "Humans") AND (MH "Animals"))) | Search modes - Boolean/Phrase | Interface - EBSCOhost Research Databases  Search Screen - Advanced Search  Database - MEDLINE Complete |
| S6 | S5 NOT PT (comment or editorial or news or newspaper article) | Search modes - Boolean/Phrase | Interface - EBSCOhost Research Databases  Search Screen - Advanced Search  Database - MEDLINE Complete |
| S5 | S3 AND S4 | Search modes - Boolean/Phrase | Interface - EBSCOhost Research Databases  Search Screen - Advanced Search  Database - MEDLINE Complete |
| S4 | PT ("systematic review" OR "meta analysis") or MH ("systematic reviews as topic+" OR "meta-analysis as topic+" OR " Technology assessment, biomedical+") or TI (meta-analy* or metanaly* or metaanaly* or met analy* or integrative research or integrative review* or integrative overview* or research integration or research overview* or collaborative review* or systematic review* or systematic overview* or evidence-based review* or evidence-based overview* or (evidence N2 (review* or overview*)) or meta-review* or meta-overview* or meta-synthes* or rapid review* or "review of reviews" or umbrella review? or technology assessment* or HTA or HTAs or (network N0 (meta-analy* or metanaly* or metaanaly* or met analy*)) or (network N0 (MA or MAs)) or NMA or NMAs or MTC or MTCs or MAIC or MAICs or "indirect* compar*" or (indirect treatment* N0 compar*) or (mixed treatment* N0 compar*) or (multiple treatment* N0 compar*) or (multi-treatment* N0 compar*) or "simultaneous* compar*" or "mixed comparison#") or AB (meta-analy* or metanaly* or metaanaly* or met analy* or integrative research or integrative review* or integrative overview* or research integration or research overview* or collaborative review* or systematic review* or systematic overview* or evidence-based review* or evidence-based overview* or (evidence N2 (review* or overview*)) or meta-review* or meta-overview* or meta-synthes* or rapid review* or "review of reviews" or umbrella review? or technology assessment* or HTA or HTAs or (network N0 (meta-analy* or metanaly* or metaanaly* or met analy*)) or (network N0 (MA or MAs)) or NMA or NMAs or MTC or MTCs or MAIC or MAICs or "indirect* compar*" or (indirect treatment* N0 compar*) or (mixed treatment* N0 compar*) or (multiple treatment* N0 compar*) or (multi-treatment* N0 compar*) or "simultaneous* compar*" or "mixed comparison#") or CI (meta-analy* or metanaly* or metaanaly* or met analy* or integrative research or integrative review* or integrative overview* or research integration or research overview* or collaborative review* or systematic review* or systematic overview* or evidence-based review* or evidence-based overview* or (evidence N2 (review* or overview*)) or meta-review* or meta-overview* or meta-synthes* or rapid review* or "review of reviews" or umbrella review? or technology assessment* or HTA or HTAs or (network N0 (meta-analy* or metanaly* or metaanaly* or met analy*)) or (network N0 (MA or MAs)) or NMA or NMAs or MTC or MTCs or MAIC or MAICs or "indirect* compar*" or (indirect treatment* N0 compar*) or (mixed treatment* N0 compar*) or (multiple treatment* N0 compar*) or (multi-treatment* N0 compar*) or "simultaneous* compar*" or "mixed comparison#") or SO (cochrane or health technology assessment or evidence report or systematic reviews) | Search modes - Boolean/Phrase | Interface - EBSCOhost Research Databases  Search Screen - Advanced Search  Database - MEDLINE Complete |
| S3 | S1 OR S2 | Search modes - Boolean/Phrase | Interface - EBSCOhost Research Databases  Search Screen - Advanced Search  Database - MEDLINE Complete |
| S2 | TI (vegetarian* OR vegan* OR "plant-based" OR plantbased OR (plant* N2 diet*)) OR AB (vegetarian* OR vegan* OR "plant-based" OR plantbased OR (plant* N2 diet*)) OR CI (vegetarian* OR vegan* OR "plant-based" OR plantbased OR (plant* N2 diet*)) | Search modes - Boolean/Phrase | Interface - EBSCOhost Research Databases  Search Screen - Advanced Search  Database - MEDLINE Complete |
| S1 | MH (Vegetarians OR Vegans OR "Diet, Vegetarian" OR "Diet, Vegan") | Search modes - Boolean/Phrase | Interface - EBSCOhost Research Databases  Search Screen - Advanced Search  Database - MEDLINE Complete |
| **CINAHL (Ebsco)** | | | |
| S9 | S8 AND DT 20180101-20240101 | Search modes - Boolean/Phrase | Interface - EBSCOhost Research Databases  Search Screen - Advanced Search  Database - CINAHL Complete |
| S8 | S7 AND LA English | Search modes - Boolean/Phrase | Interface - EBSCOhost Research Databases  Search Screen - Advanced Search  Database - CINAHL Complete |
| S7 | S6 NOT (MH "Animals+") NOT ((MH "Human") AND (MH "Animals+")) | Search modes - Boolean/Phrase | Interface - EBSCOhost Research Databases  Search Screen - Advanced Search  Database - CINAHL Complete |
| S6 | S5 NOT (PT commentary OR PT letter OR PT editorial) | Search modes - Boolean/Phrase | Interface - EBSCOhost Research Databases  Search Screen - Advanced Search  Database - CINAHL Complete |
| S5 | S3 AND S4 | Search modes - Boolean/Phrase | Interface - EBSCOhost Research Databases  Search Screen - Advanced Search  Database - CINAHL Complete |
| S4 | MH ("Meta Analysis" OR "Systematic Review") OR TI ((systematic* N2 (review* or overview*)) or (methodologic* N2 (review* or overview*))) OR ((quantitative N2 (review* or overview* or synthes*)) or (research N2 (integrati* or overview*))) OR ((integrative N2 (review* or overview*)) or (collaborative N2 (review* or overview*)) or (pool* N2 analy*)) OR ("data synthes*" or "data extraction*" or "data abstraction*") OR (handsearch* or "hand search*") OR ("mantel haenszel" or peto or "der simonian" or dersimonian or "fixed effect*" or "latin square*") OR ("met analy*" or metanaly* or "technology assessment*" or HTA or HTAs or "technology overview*" or "technology appraisal*") OR ("meta regression*" or metaregression*) OR ("meta-analy*" or metaanaly* or "systematic review*" or "biomedical technology assessment*" or "bio-medical technology assessment*") OR (medline or cochrane or pubmed or medlars or embase or cinahl) OR (comparative N2 (efficacy or effectiveness)) OR ("outcomes research" or "relative effectiveness") OR ((indirect or "indirect treatment" or "mixed-treatment") N0 comparison*) OR AB ((systematic* N2 (review* or overview*)) or (methodologic* N2 (review* or overview*))) OR ((quantitative N2 (review* or overview* or synthes*)) or (research N2 (integrati* or overview*))) OR ((integrative N2 (review* or overview*)) or (collaborative N2 (review* or overview*)) or (pool* N2 analy*)) OR ("data synthes*" or "data extraction*" or "data abstraction*") OR (handsearch* or "hand search*") OR ("mantel haenszel" or peto or "der simonian" or dersimonian or "fixed effect*" or "latin square*") OR ("met analy*" or metanaly* or "technology assessment*" or HTA or HTAs or "technology overview*" or "technology appraisal*") OR ("meta regression*" or metaregression*) OR ("meta-analy*" or metaanaly* or "systematic review*" or "biomedical technology assessment*" or "bio-medical technology assessment*") OR (medline or cochrane or pubmed or medlars or embase or cinahl) OR (comparative N2 (efficacy or effectiveness)) OR ("outcomes research" or "relative effectiveness") OR ((indirect or "indirect treatment" or "mixed-treatment") N0 comparison*) | Search modes - Boolean/Phrase | Interface - EBSCOhost Research Databases  Search Screen - Advanced Search  Database - CINAHL Complete |
| S3 | S1 OR S2 | Search modes - Boolean/Phrase | Interface - EBSCOhost Research Databases  Search Screen - Advanced Search  Database - CINAHL Complete |
| S2 | TI (vegetarian* OR vegan* OR "plant-based" OR plantbased OR (plant* N2 diet*)) OR AB (vegetarian* OR vegan* OR "plant-based" OR plantbased OR (plant* N2 diet*)) | Search modes - Boolean/Phrase | Interface - EBSCOhost Research Databases  Search Screen - Advanced Search  Database - CINAHL Complete |
| S1 | MH ("Vegetarianism" OR "Plant-Based Diet") | Search modes - Boolean/Phrase | Interface - EBSCOhost Research Databases  Search Screen - Advanced Search  Database - CINAHL Complete |
| **Cochrane Database of Systematic Reviews (Ebsco)** | | | |
| S4 | S3 AND DT 2018-2024 | Search modes - Boolean/Phrase | Interface - EBSCOhost Research Databases  Search Screen - Advanced Search  Database - Cochrane Database of Systematic Reviews |
| S3 | S1 OR S2 | Search modes - Boolean/Phrase | Interface - EBSCOhost Research Databases  Search Screen - Advanced Search  Database - Cochrane Database of Systematic Reviews |
| S2 | TI (vegetarian* OR vegan* OR "plant-based" OR plantbased OR (plant* N2 diet*)) OR AB (vegetarian* OR vegan* OR "plant-based" OR plantbased OR (plant* N2 diet*)) | Search modes - Boolean/Phrase | Interface - EBSCOhost Research Databases  Search Screen - Advanced Search  Database - Cochrane Database of Systematic Reviews |
| S1 | MH (Vegetarians OR Vegans OR "Diet, Vegetarian" OR "Diet, Vegan") | Search modes - Boolean/Phrase | Interface - EBSCOhost Research Databases  Search Screen - Advanced Search  Database - Cochrane Database of Systematic Reviews |
| **Food Science Source (Ebsco)** | | | |
| S7 | S6 AND DT 20180101-20240101 | Search modes - Boolean/Phrase | Interface - EBSCOhost Research Databases  Search Screen - Advanced Search  Database - Food Science Source |
| S6 | S5 AND LA English | Search modes - Boolean/Phrase | Interface - EBSCOhost Research Databases  Search Screen - Advanced Search  Database - Food Science Source |
| S5 | S3 AND S4 | Search modes - Boolean/Phrase | Interface - EBSCOhost Research Databases  Search Screen - Advanced Search  Database - Food Science Source |
| S4 | DE "META-analysis" OR DE "SYSTEMATIC reviews" OR TI ((systematic* N2 (review* or overview*)) or (methodologic* N2 (review* or overview*))) OR ((quantitative N2 (review* or overview* or synthes*)) or (research N2 (integrati* or overview*))) OR ((integrative N2 (review* or overview*)) or (collaborative N2 (review* or overview*)) or (pool* N2 analy*)) OR ("data synthes*" or "data extraction*" or "data abstraction*") OR (handsearch* or "hand search*") OR ("mantel haenszel" or peto or "der simonian" or dersimonian or "fixed effect*" or "latin square*") OR ("met analy*" or metanaly* or "technology assessment*" or HTA or HTAs or "technology overview*" or "technology appraisal*") OR ("meta regression*" or metaregression*) OR ("meta-analy*" or metaanaly* or "systematic review*" or "biomedical technology assessment*" or "bio-medical technology assessment*") OR (medline or cochrane or pubmed or medlars or embase or cinahl) OR (comparative N2 (efficacy or effectiveness)) OR ("outcomes research" or "relative effectiveness") OR ((indirect or "indirect treatment" or "mixed-treatment") N0 comparison*) OR AB ((systematic* N2 (review* or overview*)) or (methodologic* N2 (review* or overview*))) OR ((quantitative N2 (review* or overview* or synthes*)) or (research N2 (integrati* or overview*))) OR ((integrative N2 (review* or overview*)) or (collaborative N2 (review* or overview*)) or (pool* N2 analy*)) OR ("data synthes*" or "data extraction*" or "data abstraction*") OR (handsearch* or "hand search*") OR ("mantel haenszel" or peto or "der simonian" or dersimonian or "fixed effect*" or "latin square*") OR ("met analy*" or metanaly* or "technology assessment*" or HTA or HTAs or "technology overview*" or "technology appraisal*") OR ("meta regression*" or metaregression*) OR ("meta-analy*" or metaanaly* or "systematic review*" or "biomedical technology assessment*" or "bio-medical technology assessment*") OR (medline or cochrane or pubmed or medlars or embase or cinahl) OR (comparative N2 (efficacy or effectiveness)) OR ("outcomes research" or "relative effectiveness") OR ((indirect or "indirect treatment" or "mixed-treatment") N0 comparison*) OR KW ((systematic* N2 (review* or overview*)) or (methodologic* N2 (review* or overview*))) OR ((quantitative N2 (review* or overview* or synthes*)) or (research N2 (integrati* or overview*))) OR ((integrative N2 (review* or overview*)) or (collaborative N2 (review* or overview*)) or (pool* N2 analy*)) OR ("data synthes*" or "data extraction*" or "data abstraction*") OR (handsearch* or "hand search*") OR ("mantel haenszel" or peto or "der simonian" or dersimonian or "fixed effect*" or "latin square*") OR ("met analy*" or metanaly* or "technology assessment*" or HTA or HTAs or "technology overview*" or "technology appraisal*") OR ("meta regression*" or metaregression*) OR ("meta-analy*" or metaanaly* or "systematic review*" or "biomedical technology assessment*" or "bio-medical technology assessment*") OR (medline or cochrane or pubmed or medlars or embase or cinahl) OR (comparative N2 (efficacy or effectiveness)) OR ("outcomes research" or "relative effectiveness") OR ((indirect or "indirect treatment" or "mixed-treatment") N0 comparison*) OR SO (cochrane or health technology assessment or evidence report or systematic reviews) | Search modes - Boolean/Phrase | Interface - EBSCOhost Research Databases  Search Screen - Advanced Search  Database - Food Science Source |
| S3 | S1 OR S2 | Search modes - Boolean/Phrase | Interface - EBSCOhost Research Databases  Search Screen - Advanced Search  Database - Food Science Source |
| S2 | TI (vegetarian* OR vegan* OR "plant-based" OR plantbased OR (plant* N2 diet*)) OR AB (vegetarian* OR vegan* OR "plant-based" OR plantbased OR (plant* N2 diet*)) OR KW (vegetarian* OR vegan* OR "plant-based" OR plantbased OR (plant* N2 diet*)) | Search modes - Boolean/Phrase | Interface - EBSCOhost Research Databases  Search Screen - Advanced Search  Database - Food Science Source |
| S1 | DE "VEGETARIANISM" OR DE "LACTO-ovo vegetarianism" OR DE "VEGANISM" OR DE "VEGETARIAN cooking" OR DE "VEGETARIANS" OR DE "VEGANS" OR DE "VEGANISM" OR DE "VEGAN cooking" OR DE "PLANT-based diet" | Search modes - Boolean/Phrase | Interface - EBSCOhost Research Databases  Search Screen - Advanced Search  Database - Food Science Source |
| **SportsDiscus (Ebsco)** | | | |
| S7 | S6 AND DT 20180101-20240101 | Search modes - Boolean/Phrase | Interface - EBSCOhost Research Databases  Search Screen - Advanced Search  Database - SPORTDiscus with Full Text |
| S6 | S5 AND LA English | Search modes - Boolean/Phrase | Interface - EBSCOhost Research Databases  Search Screen - Advanced Search  Database - SPORTDiscus with Full Text |
| S5 | S3 AND S4 | Search modes - Boolean/Phrase | Interface - EBSCOhost Research Databases  Search Screen - Advanced Search  Database - SPORTDiscus with Full Text |
| S4 | TI ((systematic* N2 (review* or overview*)) or (methodologic* N2 (review* or overview*))) OR ((quantitative N2 (review* or overview* or synthes*)) or (research N2 (integrati* or overview*))) OR ((integrative N2 (review* or overview*)) or (collaborative N2 (review* or overview*)) or (pool* N2 analy*)) OR ("data synthes*" or "data extraction*" or "data abstraction*") OR (handsearch* or "hand search*") OR ("mantel haenszel" or peto or "der simonian" or dersimonian or "fixed effect*" or "latin square*") OR ("met analy*" or metanaly* or "technology assessment*" or HTA or HTAs or "technology overview*" or "technology appraisal*") OR ("meta regression*" or metaregression*) OR ("meta-analy*" or metaanaly* or "systematic review*" or "biomedical technology assessment*" or "bio-medical technology assessment*") OR (medline or cochrane or pubmed or medlars or embase or cinahl) OR (comparative N2 (efficacy or effectiveness)) OR ("outcomes research" or "relative effectiveness") OR ((indirect or "indirect treatment" or "mixed-treatment") N0 comparison*) OR AB ((systematic* N2 (review* or overview*)) or (methodologic* N2 (review* or overview*))) OR ((quantitative N2 (review* or overview* or synthes*)) or (research N2 (integrati* or overview*))) OR ((integrative N2 (review* or overview*)) or (collaborative N2 (review* or overview*)) or (pool* N2 analy*)) OR ("data synthes*" or "data extraction*" or "data abstraction*") OR (handsearch* or "hand search*") OR ("mantel haenszel" or peto or "der simonian" or dersimonian or "fixed effect*" or "latin square*") OR ("met analy*" or metanaly* or "technology assessment*" or HTA or HTAs or "technology overview*" or "technology appraisal*") OR ("meta regression*" or metaregression*) OR ("meta-analy*" or metaanaly* or "systematic review*" or "biomedical technology assessment*" or "bio-medical technology assessment*") OR (medline or cochrane or pubmed or medlars or embase or cinahl) OR (comparative N2 (efficacy or effectiveness)) OR ("outcomes research" or "relative effectiveness") OR ((indirect or "indirect treatment" or "mixed-treatment") N0 comparison*) OR KW ((systematic* N2 (review* or overview*)) or (methodologic* N2 (review* or overview*))) OR ((quantitative N2 (review* or overview* or synthes*)) or (research N2 (integrati* or overview*))) OR ((integrative N2 (review* or overview*)) or (collaborative N2 (review* or overview*)) or (pool* N2 analy*)) OR ("data synthes*" or "data extraction*" or "data abstraction*") OR (handsearch* or "hand search*") OR ("mantel haenszel" or peto or "der simonian" or dersimonian or "fixed effect*" or "latin square*") OR ("met analy*" or metanaly* or "technology assessment*" or HTA or HTAs or "technology overview*" or "technology appraisal*") OR ("meta regression*" or metaregression*) OR ("meta-analy*" or metaanaly* or "systematic review*" or "biomedical technology assessment*" or "bio-medical technology assessment*") OR (medline or cochrane or pubmed or medlars or embase or cinahl) OR (comparative N2 (efficacy or effectiveness)) OR ("outcomes research" or "relative effectiveness") OR ((indirect or "indirect treatment" or "mixed-treatment") N0 comparison*) OR SO (cochrane or health technology assessment or evidence report or systematic reviews) | Search modes - Boolean/Phrase | Interface - EBSCOhost Research Databases  Search Screen - Advanced Search  Database - SPORTDiscus with Full Text |
| S3 | S1 OR S2 | Search modes - Boolean/Phrase | Interface - EBSCOhost Research Databases  Search Screen - Advanced Search  Database - SPORTDiscus with Full Text |
| S2 | TI (vegetarian* OR vegan* OR "plant-based" OR plantbased OR (plant* N2 diet*)) OR AB (vegetarian* OR vegan* OR "plant-based" OR plantbased OR (plant* N2 diet*)) OR KW (vegetarian* OR vegan* OR "plant-based" OR plantbased OR (plant* N2 diet*)) | Search modes - Boolean/Phrase | Interface - EBSCOhost Research Databases  Search Screen - Advanced Search  Database - SPORTDiscus with Full Text |
| S1 | DE "VEGETARIANISM" OR DE "VEGANISM" OR DE "PLANT-based diet" | Search modes - Boolean/Phrase | Interface - EBSCOhost Research Databases  Search Screen - Advanced Search  Database - SPORTDiscus with Full Text |

**Supplementary Table 2. List of Systematic Reviews Excluded in the Umbrella Review Examining the Research Question: In presumably healthy adults in the general population, what is the relationship between vegetarian diets, compared to non-vegetarian diets, on outcomes of interest?**

| **Articles** | **Reason for Exclusion** |
| --- | --- |
| 1. Aleksandrova K, Koelman L, Rodrigues CE. Dietary patterns and biomarkers of oxidative stress and inflammation: A systematic review of observational and intervention studies. *Redox Biol*. 2021;42:101869. doi:10.1016/j.redox.2021.101869 PMID: 33541846 | No outcome of interest |
| 1. Amiri M, Karabegović I, van Westing AC, et al. Whole-diet interventions and cardiovascular risk factors in postmenopausal women: A systematic review of controlled clinical trials. *Maturitas*. 2022;155:40-53. doi:10.1016/j.maturitas.2021.10.001. PMID: 34876248 | Wrong Exposure/Intervention |
| 1. Carroll KL, Frugé AD, Heslin MJ, Lipke EA, Greene MW. Diet as a Risk Factor for Early-Onset Colorectal Adenoma and Carcinoma: A Systematic Review. *Front Nutr*. 2022;9:896330. Published 2022 Jun 9. doi:10.3389/fnut.2022.896330. PMID: 35757246 | Wrong Exposure/Intervention |
| 1. Chan H, Ribeiro RV, Haden S, Hirani V. Plant-Based Dietary Patterns, Body Composition, Muscle Strength and Function in Middle and Older Age: A Systematic Review. *J Nutr Health Agin*g. 2021;25(8):1012-1022. doi:10.1007/s12603-021-1666-7. PMID: 34545922 | No outcome of interest |
| 1. Chareonrungrueangchai K, Wongkawinwoot K, Anothaisintawee T, Reutrakul S. Dietary Factors and Risks of Cardiovascular Diseases: An Umbrella Review. *Nutrients*. 2020;12(4):1088. Published 2020 Apr 15. doi:10.3390/nu12041088. PMID: 32326404 | Other: Umbrella Review |
| 1. Chhabra JS, Juneja A, Etkin Y, et al. The effect of nutrition on stroke risk: A systematic review. *Nutr Health*. 2023;29(2):255-267. doi:10.1177/02601060221122218. PMID: 36040714 | Searched < 2 databases |
| 1. English LK, Ard JD, Bailey RL, et al. Evaluation of Dietary Patterns and All-Cause Mortality: A Systematic Review. *JAMA Netw Open*. 2021;4(8):e2122277. Published 2021 Aug 2. doi:10.1001/jamanetworkopen.2021.22277. PMID: 34463743 | Wrong Exposure/Intervention |
| 1. Eveleigh ER, Coneyworth LJ, Avery A, Welham SJM. Vegans, Vegetarians, and Omnivores: How Does Dietary Choice Influence Iodine Intake? A Systematic Review. *Nutrients*. 2020;12(6):1606. Published 2020 May 29. doi:10.3390/nu12061606. PMID: 32486114 | No outcome of interest |
| 1. Fan M, Li Y, Wang C, et al. Dietary Protein Consumption and the Risk of Type 2 Diabetes: ADose-Response Meta-Analysis of Prospective Studies. *Nutrients*. 2019;11(11):2783. Published 2019 Nov 15. doi:10.3390/nu11112783. PMID: 31731672 | Wrong Exposure/Intervention |
| 1. Fontes T, Rodrigues LM, Ferreira-Pêgo C. Comparison between Different Groups of Vegetarianism and Its Associations with Body Composition: A Literature Review from 2015 to 2021. *Nutrients*. 2022;14(9):1853. Published 2022 Apr 28. doi:10.3390/nu14091853. PMID: 35565820 | Searched < 2 databases |
| 1. Gan ZH, Cheong HC, Tu YK, Kuo PH. Association between Plant-Based Dietary Patterns and Risk of Cardiovascular Disease: A Systematic Review and Meta-Analysis of Prospective Cohort Studies. *Nutrients*. 2021;13(11):3952. Published 2021 Nov 5. doi:10.3390/nu13113952. PMID: 34836208 | Wrong Exposure/Intervention |
| 1. Guasch-Ferré M, Satija A, Blondin SA, et al. Meta-Analysis of Randomized Controlled Trials of Red Meat Consumption in Comparison With Various Comparison Diets on Cardiovascular Risk Factors. *Circulation*. 2019;139(15):1828-1845. doi:10.1161/CIRCULATIONAHA.118.035225. PMID: 30957819 | Wrong Exposure/Intervention |
| 1. Gupta N, Patel HD, Taylor J, et al. Systematic review of the impact of a plant-based diet on prostate cancer incidence and outcomes. *Prostate Cancer Prostatic Dis*. 2022;25(3):444-452. doi:10.1038/s41391-022-00553-2. PMID: 35790788 | Wrong Exposure/Intervention |
| 1. Haider LM, Schwingshackl L, Hoffmann G, Ekmekcioglu C. The effect of vegetarian diets on iron status in adults: A systematic review and meta-analysis. *Crit Rev Food Sci Nutr*. 2018;58(8):1359-1374. doi:10.1080/10408398.2016.1259210. PMID: 27880062 | No outcome of interest |
| 1. Ibrahim MO, Abuhijleh H, Tayyem R. What Dietary Patterns and Nutrients are Associated with Pancreatic Cancer? Literature Review. *Cancer Manag Res*. 2023;15:17-30. Published 2023 Jan 6. doi:10.2147/CMAR.S390228. PMID: 36643074 | Wrong Exposure/Intervention |
| 1. Iguacel I, Miguel-Berges ML, Gómez-Bruton A, Moreno LA, Julián C. Veganism, vegetarianism, bone mineral density, and fracture risk: a systematic review and meta-analysis. *Nutr Rev*. 2019;77(1):1-18. doi:10.1093/nutrit/nuy045. PMID: 30376075 | No outcome of interest |
| 1. Janko RK, Wilson P, Nworie C. The effects of plant-based diets on pancreatic beta-cell function: A systematic review. J Diabetes Nurs. 2021;25(2):1-7. | No outcome of interest |
| 1. Jarvis SE, Nguyen M, Malik VS. Association between adherence to plant-based dietary patterns and obesity risk: a systematic review of prospective cohort studies. *Appl Physiol Nutr Metab*. 2022;47(12):1115-1133. doi:10.1139/apnm-2022-0059> PMID: 35985038 | Wrong Exposure/Intervention |
| 1. Jiang K, Zhang Z, Fullington LA, et al. Dietary Patterns and Obesity in Chinese Adults: A Systematic Review and Meta-Analysis. *Nutrients*. 2022;14(22):4911. Published 2022 Nov 20. doi:10.3390/nu14224911. PMID: 36432596 | Wrong Exposure/Intervention |
| 1. Johannesen CO, Dale HF, Jensen C, Lied GA. Effects of Plant-Based Diets on Outcomes Related to Glucose Metabolism: A Systematic Review. *Diabetes Metab Syndr Obes*. 2020;13:2811-2822. Published 2020 Aug 7. doi:10.2147/DMSO.S265982. PMID: 322884310 | Searched < 2 databases |
| 1. Koelman L, Egea Rodrigues C, Aleksandrova K. Effects of Dietary Patterns on Biomarkers of Inflammation and Immune Responses: A Systematic Review and Meta-Analysis of Randomized Controlled Trials. *Adv Nutr*. 2022;13(1):101-115. doi:10.1093/advances/nmab086. PMID: 34607347 | Wrong Exposure/Intervention |
| 1. Koutras Y, Chrysostomou S, Poulimeneas D, Yannakoulia M. Examining the associations between a posteriori dietary patterns and obesity indexes: Systematic review of observational studies. *Nutr Health*. 2022;28(2):149-162. doi:10.1177/02601060211020975. PMID: 34100659 | Wrong Exposure/Intervention |
| 1. Lamberg-Allardt C, Barebring L, Arnesen EK, Nwaru BI, Thorisdorrir B, Ramel A, et al. Animal versus plant-based protein and risk of cardiovascular disease and type 2 diabetes: a systematic review of randomized controlled trials and prospective cohort studies. *Food Nutr Res.* 2023;28:67. | Wrong Exposure/Intervention |
| 1. Li J, Zhou R, Huang W, Wang J. Bone loss, low height, and low weight in different populations and district: a meta-analysis between vegans and non-vegans. *Food Nutr Res*. 2020;64:10.29219/fnr.v64.3315. Published 2020 Sep 11. doi:10.29219/fnr.v64.3315. PMID: 33061885 | No outcome of interest |
| 1. Li T, Li Y, Wu S. Comparison of human bone mineral densities in subjects on plant-based and omnivorous diets: a systematic review and meta-analysis. *Arch Osteoporos*. 2021;16(1):95. Published 2021 Jun 18. doi:10.1007/s11657-021-00955-0. PMID: 34145511 | No outcome of interest |
| 1. Liang S, Mijatovic J, Li A, et al. Dietary Patterns and Non-Communicable Disease Biomarkers: A Network Meta-Analysis and Nutritional Geometry Approach. *Nutrients*. 2022;15(1):76. Published 2022 Dec 23. doi:10.3390/nu15010076. PMID: 36615733 | Wrong Exposure/Intervention |
| 1. Lim MT, Pan BJ, Toh DWK, Sutanto CN, Kim JE. Animal Protein versus Plant Protein in Supporting Lean Mass and Muscle Strength: A Systematic Review and Meta-Analysis of Randomized Controlled Trials. *Nutrients*. 2021;13(2):661. Published 2021 Feb 18. doi:10.3390/nu13020661. PMID: 33670701 | Wrong Exposure/Intervention |
| 1. Liu Y, Zhu L, Li D, Wang L, Tang H, Zhang C. Stroke risk with vegetarian, low-animal and high-animal diets: A systematic review and meta-analysis. *Asia Pac J Clin Nutr*. 2022;31(3):422-432. doi:10.6133/apjcn.202209_31(3).0010. PMID: 36173214 | No Comparison |
| 1. Long Y, Ye H, Yang J, Tao X, Xie H, Zhang J. Effects of a vegetarian diet combined with aerobic exercise on glycemic control, insulin resistance, and body composition: a systematic review and meta-analysis. Eat Weight Disord. 2023;28(1):9. | Wrong Exposure/Intervention |
| 1. Lopes T, Zemlin AE, Erasmus RT, Madlala SS, Faber M, Kengne AP. Assessment of the association between plant-based dietary exposures and cardiovascular disease risk profile in sub-Saharan Africa: a systematic review. *BMC Public Health*. 2022;22(1):361. Published 2022 Feb 19. doi:10.1186/s12889-022-12724-w. PMID: 35183139 | Wrong Exposure/Intervention |
| 1. Ma X, Tan H, Hu M, He S, Zou L, Pan H. The impact of plant-based diets on female bone mineral density: Evidence based on seventeen studies. *Medicine (Baltimore)*. 2021;100(46):e27480. doi:10.1097/MD.0000000000027480. PMID: 34797275 | No outcome of interest |
| 1. Medawar E, Huhn S, Villringer A, Veronica Witte A. The effects of plant-based diets on the body and the brain: a systematic review. *Transl Psychiatry*. 2019;9(1):226. Published 2019 Sep 12. doi:10.1038/s41398-019-0552-0. PMID: 31515473 | Searched < 2 databases |
| 1. Medina-Remón A, Kirwan R, Lamuela-Raventós RM, Estruch R. Dietary patterns and the risk of obesity, type 2 diabetes mellitus, cardiovascular diseases, asthma, and neurodegenerative diseases. *Crit Rev Food Sci Nutr*. 2018;58(2):262-296. doi:10.1080/10408398.2016.1158690. PMID: 27127938 | Wrong Exposure/Intervention |
| 1. Molina-Montes E, Salamanca-Fernández E, Garcia-Villanova B, Sánchez MJ. The Impact of Plant-Based Dietary Patterns on Cancer-Related Outcomes: A Rapid Review and Meta-Analysis. *Nutrients*. 2020;12(7):2010. Published 2020 Jul 6. doi:10.3390/nu12072010. PMID: 32640737 | Searched < 2 databases |
| 1. Molina-Montes E, Ubago-Guisado E, Petrova D, et al. The Role of Diet, Alcohol, BMI, and Physical Activity in Cancer Mortality: Summary Findings of the EPIC Study. *Nutrients*. 2021;13(12):4293. Published 2021 Nov 28. doi:10.3390/nu13124293. PMID: 34959845 | Wrong Population |
| 1. Mousavi SM, Jayedi A, Jalilpiran Y, Hajishafiee M, Aminianfar A, Esmaillzadeh A. Dietary intake of total, animal and plant proteins and the risk of coronary heart disease and hypertension: a systematic review and dose-response meta-analysis of prospective cohort studies. *Crit Rev Food Sci Nutr*. 2022;62(5):1336-1349. doi:10.1080/10408398.2020.1841730. PMID: 33131293 | Wrong Exposure/Intervention |
| 1. Neufingerl N, Eilander A. Nutrient Intake and Status in Adults Consuming Plant-Based Diets Compared to Meat-Eaters: A Systematic Review. *Nutrients*. 2021;14(1):29. Published 2021 Dec 23. doi:10.3390/nu14010029. PMID: 3501904 | Searched < 2 databases |
| 1. Niu Y, Cao H, Zhou H, Cao J, Wang Z. Effects of a vegetarian diet combined with exercise on lipid profiles and blood pressure: A systematic review and meta-analysis [published online ahead of print, 2022 Sep 15]. *Crit Rev Food Sci Nutr*. 2022;1-15. doi:10.1080/10408398.2022.2122923. PMID: 36106474 | Wrong Exposure/Intervention |
| 1. Parra-Soto S, Ahumada D, Petermann-Rocha F, et al. Association of meat, vegetarian, pescatarian and fish-poultry diets with risk of 19 cancer sites and all cancer: findings from the UK Biobank prospective cohort study and meta-analysis. *BMC Med*. 2022;20(1):79. Published 2022 Feb 24. doi:10.1186/s12916-022-02257-9. PMID: 35655214 | No outcome of interest |
| 1. Picasso MC, Lo-Tayraco JA, Ramos-Villanueva JM, Pasupuleti V, Hernandez AV. Effect of vegetarian diets on the presentation of metabolic syndrome or its components: A systematic review and meta-analysis. *Clin Nutr*. 2019;38(3):1117-1132. doi:10.1016/j.clnu.2018.05.021. PMID: 29907356 | Wrong Exposure/Intervention |
| 1. Poulsen NB, Lambert MNT, Jeppesen PB. The Effect of Plant Derived Bioactive Compounds on Inflammation: A Systematic Review and Meta-Analysis. *Mol Nutr Food Res*. 2020;64(18):e2000473. doi:10.1002/mnfr.202000473. PMID: 32761736 | Wrong Exposure/Intervention |
| 1. Qi XX, Shen P. Associations of dietary protein intake with all-cause, cardiovascular disease, and cancer mortality: A systematic review and meta-analysis of cohort studies. *Nutr Metab Cardiovasc Dis*. 2020;30(7):1094-1105. doi:10.1016/j.numecd.2020.03.008. PMID: 32451273 | Wrong Exposure/Intervention |
| 1. Qian F, Liu G, Hu FB, Bhupathiraju SN, Sun Q. Association Between Plant-Based Dietary Patterns and Risk of Type 2 Diabetes: A Systematic Review and Meta-analysis. *JAMA Intern Med*. 2019;179(10):1335-1344. doi:10.1001/jamainternmed.2019.2195. PMID: 31329220 | Wrong Exposure/Intervention |
| 1. Salehiniya H, Bahadori M, Ghanizadeh G, Raei M. Epidemiological Study of Lung Cancer in Iran: A Systematic Review. *Iran J Public Health*. 2022;51(2):306-317. doi:10.18502/ijph.v51i2.8683. PMID: 35866136 | Wrong Exposure/Intervention |
| 1. Sanches Machado d'Almeida K, Ronchi Spillere S, Zuchinali P, Corrêa Souza G. Mediterranean Diet and Other Dietary Patterns in Primary Prevention of Heart Failure and Changes in Cardiac Function Markers: A Systematic Review. Nutrients. 2018;10(1):58. Published 2018 Jan 10. doi:10.3390/nu10010058. PMID: 29320401 | No outcome of interest |
| 1. Saz-Lara A, Battino M, Del Saz L, Cavero-Redondo IDavalos A, Lopez de Las Hazas MC, et al. Differences in carotid to femoral pulse wave velocity and carotid intima media thickness between vegetarian and omnivorous diets in healthy subjects: a systematic review and meta-analysis. Food Funct. 2024;15(3):1135-1143. | No outcome of interest |
| 1. Seidelmann SB, Claggett B, Cheng S, et al. Dietary carbohydrate intake and mortality: a prospective cohort study and meta-analysis. Lancet Public Health. 2018;3(9):e419-e428. doi:10.1016/S2468-2667(18)30135-X. PMID: 30122560 | Wrong Exposure/Intervention |
| 1. Shin J, Millstine D, Ruddy B, Wallace M, Fields H. Effect of Plant- and Animal-Based Foods on Prostate Cancer Risk [published online ahead of print, 2019 Oct 21]. J Am Osteopath Assoc. 2019;10.7556/jaoa.2019.123. doi:10.7556/jaoa.2019.123. PMID: 31633743 | No exposure of interest |
| 1. Siqueira CHIA, Esteves LG, Duarte CK. Plant-based diet index score is not associated with body composition: A systematic review and meta-analysis. Nutr Res. 2022;104:128-139. doi:10.1016/j.nutres.2022.05.005. PMID: 35763983 | Wrong Exposure/Intervention |
| 1. Sluik D, Brouwer-Brolsma EM, Berendsen AAM, et al. Protein intake and the incidence of pre-diabetes and diabetes in 4 population-based studies: the PREVIEW project. Am J Clin Nutr. 2019;109(5):1310-1318. doi:10.1093/ajcn/nqy388. PMID: 31051510 | Wrong Population |
| 1. Stoodley IL, Williams LM, Wood LG. Effects of Plant-Based Protein Interventions, with and without an Exercise Component, on Body Composition, Strength and Physical Function in Older Adults: A Systematic Review and Meta-Analysis of Randomized Controlled Trials. Nutrients. 15(18):4060. | Wrong Exposure/Intervention |
| 1. Termannsen AD, Clemmensen KKB, Thomsen JM, et al. Effects of vegan diets on cardiometabolic health: A systematic review and meta-analysis of randomized controlled trials. Obes Rev. 2022;23(9):e13462. doi:10.1111/obr.13462. PMID: 35672940 | Wrong Population |
| 1. [Tomé-Carneiro](https://pubmed.ncbi.nlm.nih.gov/?term=Tom%C3%A9-Carneiro+J&cauthor_id=37178356) J, Visioli F. Plant-Based Diets Reduce Blood Pressure: A Systematic Review of Recent Evidence. Curr Hypertens Rep. 2023; 25(7):127-150. | Other: Umbrella Review |
| 1. Tran E, Dale HF, Jensen C, Lied GA. Effects of Plant-Based Diets on Weight Status: A Systematic Review. Diabetes Metab Syndr Obes. 2020;13:3433-3448. Published 2020 Sep 30. doi:10.2147/DMSO.S272802. PMID: 33061504 | Searched < 2 databases |
| 1. Tucci M, Marino M, Martini D, Porrini M, Riso P, Del Bo' C. Plant-Based Foods and Vascular Function: A Systematic Review of Dietary Intervention Trials in Older Subjects and Hypothesized Mechanisms of Action. *Nutrients*. 2022;14(13):2615. Published 2022 Jun 24. doi:10.3390/nu14132615. PMID: 35807796 | Wrong Exposure/Intervention |
| 1. Webb A, Lane K. The effectiveness of a low-fat vegan diet for the prevention and management of type 2 diabetes: A systematic review. *Proceedings of the Nutrition Society*. 2020;79(OCE2):E408. doi:10.1017/S0029665120003560. No PMID | Other |
| 1. Zhao Y, Zhan J, Wang Y, Wang D. The Relationship Between Plant-Based Diet and Risk of Digestive System Cancers: A Meta-Analysis Based on 3,059,009 Subjects. *Front Public Health*. 2022;10:892153. Published 2022 Jun 3. doi:10.3389/fpubh.2022.892153. PMID: 35719615 | Wrong Exposure/Intervention |
| 1. Zheng J, Zhu T, Yang G, et al. The Isocaloric Substitution of Plant-Based and Animal-Based Protein in Relation to Aging-Related Health Outcomes: A Systematic Review. *Nutrients*. 2022;14(2):272. Published 2022 Jan 9. doi:10.3390/nu14020272. PMID: 35057453 | Wrong Exposure/Intervention |
| 1. Zyriax BC, Windler E. Lifestyle changes to prevent cardio- and cerebrovascular disease at midlife: A systematic review. *Maturitas*. 2023;167:60-65. doi:10.1016/j.maturitas.2022.09.003. PMID: 36306668 | Wrong Exposure/Intervention |

**Supplementary Table 3. AMSTAR2 ^2^ Ratings in Critical Systematic Review Domains and Overall Certainty in Results for Systematic Reviews Included in the Umbrella Review Examining the Relationships Between a Vegetarian/Vegan Diet, Compared to a Non-Vegetarian Diet and Cardiovascular Risk and Disease Outcomes**

| **Systematic Review** | **Explicit statement of a priori review methods** | **Comprehensive literature search strategy** | **List of excluded studies with justification** | **Satisfactory technique for assessing risk of bias** | **If meta‐ analysis, appropriate methods of combining results** | **Account for risk of bias of included studies when interpreting result** | **If quantitative analysis, investigation and discussion of impact of publication bias** | **Overall Confidence in Results of Review** |
| --- | --- | --- | --- | --- | --- | --- | --- | --- |
| **Bakaloudi et al 2021^3^** | Y | Y | N | Y | No meta-analysis | Y | No meta-analysis | Low |
| **Benatar et al 2018^4^** | N | Y | N | Y | Y | N | Y | Critically Low |
| **Craddock et al 2019^5^** | Y | Y | N | Y | Y for RCT | Y | N | Critically Low |
| **Dybvik et al 2022^6^** | N | Y | Y | Y | Y | Y | Y | Low |
| **Elliot et al 2022^7^** | N | Y | N | N | No meta-analysis | N | No meta-analysis | Critically Low |
| **Gibbs et al. 2021^8^** | Y | Y | Y | Y | Y | Y | Y | Moderate |
| **Glenn et al 2019^9^** | Y | Y | N | Y | Y | N | Y | Critically Low |
| **Ivanova et al 2021^10^** | N | N | N | N | No meta-analysis | N | No meta-analysis | Critically Low |
| **Jabri et al 2021^11^** | N | Y | N | Y | Y | Y | Y | Critically Low |
| **Jafari et al 2021^12^** | N | Y | Y | Y | Y | Y | Y | Low |
| **Kaiser et al 2021^13^** | Y | Y | N | Y | No meta-analysis | Y | No meta-analysis | Low |
| **Koch et al 2023^14^** | N | Y | N | Y | Y | Y | Y | Critically Low |
| **Lee et al 2020^15^** | Y | Y | N | Y | Y | Y | Y | Critically Low |
| **Lopez et al 2019^16^** | Y | Y | N | Y | Y | Y | Y | Low |
| **Lu et al 2021^17^** | Y | Y | Y | Y | Y | Y | Y | High |
| **Menzel et al 2020^18^** | Y | Y | N | Y | Y | Y | Y | Low |
| **Pollakova et al 2021^19^** | N | Y | N | Y | No meta-analysis | N | No meta-analysis | Critically Low |
| **Quek et al 2021^20^** | Y | Y | N | Y | Y | N | Y | Critically Low |
| **Rees et al 2021^21^** | Y | Y | Y | Y | Y | Y | Y | High |
| **Remde et al 2022^22^** | PY | Y | N | Y | No meta-analysis | Y | No meta-analysis | Low |
| **Wang et al 2023^23^** | Y | Y | N | Y | Y | N | Y | Critically Low |

Abbreviations: N= No, PY= Partial Yes, RCT= randomized controlled trial, Y= Yes

Supplemental Literature Cited

1. Landry MJ, Ward CP. Health Benefits of a Plant-Based Dietary Pattern and Implementation in Healthcare and Clinical Practice. *American Journal of Lifestyle Medicine.* 2024:15598276241237766.

2. Shea BJ, Reeves BC, Wells G, et al. AMSTAR 2: a critical appraisal tool for systematic reviews that include randomised or non-randomised studies of healthcare interventions, or both. *Bmj.* 2017;358:j4008.

3. Bakaloudi DR, Halloran A, Rippin HL, et al. Intake and adequacy of the vegan diet. A systematic review of the evidence. *Clin Nutr.* 2021;40(5):3503-3521.

4. Benatar JR, Stewart RAH. Cardiometabolic risk factors in vegans; A meta-analysis of observational studies. *PLoS One.* 2018;13(12):e0209086.

5. Craddock JC, Neale EP, Peoples GE, Probst YC. Vegetarian-Based Dietary Patterns and their Relation with Inflammatory and Immune Biomarkers: A Systematic Review and Meta-Analysis. *Adv Nutr.* 2019;10(3):433-451.

6. Dybvik JS, Svendsen M, Aune D. Vegetarian and vegan diets and the risk of cardiovascular disease, ischemic heart disease and stroke: a systematic review and meta-analysis of prospective cohort studies. *Eur J Nutr.* 2023;62(1):51-69.

7. Elliott PS, Kharaty SS, Phillips CM. Plant-Based Diets and Lipid, Lipoprotein, and Inflammatory Biomarkers of Cardiovascular Disease: A Review of Observational and Interventional Studies. *Nutrients.* 2022;14(24).

8. Gibbs J, Gaskin E, Ji C, Miller MA, Cappuccio FP. The effect of plant-based dietary patterns on blood pressure: a systematic review and meta-analysis of controlled intervention trials. *J Hypertens.* 2021;39(1):23-37.

9. Glenn AJ, Viguiliouk E, Seider M, et al. Relation of Vegetarian Dietary Patterns With Major Cardiovascular Outcomes: A Systematic Review and Meta-Analysis of Prospective Cohort Studies. *Front Nutr.* 2019;6:80.

10. Ivanova S, Delattre C, Karcheva-Bahchevanska D, Benbasat N, Nalbantova V, Ivanov K. Plant-Based Diet as a Strategy for Weight Control. *Foods.* 2021;10(12).

11. Jabri A, Kumar A, Verghese E, et al. Meta-analysis of effect of vegetarian diet on ischemic heart disease and all-cause mortality. *Am J Prev Cardiol.* 2021;7:100182.

12. Jafari S, Hezaveh E, Jalilpiran Y, et al. Plant-based diets and risk of disease mortality: a systematic review and meta-analysis of cohort studies. *Crit Rev Food Sci Nutr.* 2022;62(28):7760-7772.

13. Kaiser J, van Daalen KR, Thayyil A, Cocco M, Caputo D, Oliver-Williams C. A Systematic Review of the Association Between Vegan Diets and Risk of Cardiovascular Disease. *J Nutr.* 2021;151(6):1539-1552.

14. Koch CA, Kjeldsen EW, Frikke-Schmidt R. Vegetarian or vegan diets and blood lipids: a meta-analysis of randomized trials. *Eur Heart J.* 2023;44(28):2609-2622.

15. Lee KW, Loh HC, Ching SM, Devaraj NK, Hoo FK. Effects of Vegetarian Diets on Blood Pressure Lowering: A Systematic Review with Meta-Analysis and Trial Sequential Analysis. *Nutrients.* 2020;12(6).

16. Lopez PD, Cativo EH, Atlas SA, Rosendorff C. The Effect of Vegan Diets on Blood Pressure in Adults: A Meta-Analysis of Randomized Controlled Trials. *Am J Med.* 2019;132(7):875-883.e877.

17. Lu JW, Yu LH, Tu YK, et al. Risk of Incident Stroke among Vegetarians Compared to Nonvegetarians: A Systematic Review and Meta-Analysis of Prospective Cohort Studies. *Nutrients.* 2021;13(9).

18. Menzel J, Jabakhanji A, Biemann R, Mai K, Abraham K, Weikert C. Systematic review and meta-analysis of the associations of vegan and vegetarian diets with inflammatory biomarkers. *Sci Rep.* 2020;10(1):21736.

19. Pollakova D, Andreadi A, Pacifici F, Della-Morte D, Lauro D, Tubili C. The Impact of Vegan Diet in the Prevention and Treatment of Type 2 Diabetes: A Systematic Review. *Nutrients.* 2021;13(6).

20. Quek J, Lim G, Lim WH, et al. The Association of Plant-Based Diet With Cardiovascular Disease and Mortality: A Meta-Analysis and Systematic Review of Prospect Cohort Studies. *Front Cardiovasc Med.* 2021;8:756810.

21. Rees K, Al-Khudairy L, Takeda A, Stranges S. Vegan dietary pattern for the primary and secondary prevention of cardiovascular diseases. *Cochrane Database Syst Rev.* 2021;2(2):Cd013501.

22. Remde A, DeTurk SN, Almardini A, Steiner L, Wojda T. Plant-predominant eating patterns - how effective are they for treating obesity and related cardiometabolic health outcomes? - a systematic review. *Nutr Rev.* 2022;80(5):1094-1104.

23. Wang Y, Liu B, Han H, et al. Associations between plant-based dietary patterns and risks of type 2 diabetes, cardiovascular disease, cancer, and mortality - a systematic review and meta-analysis. *Nutr J.* 2023;22(1):46.
